# Pre-Clinical Study Evaluating Novel Protein Phosphatase 2A Activators as Therapeutics for Neuroblastoma

**DOI:** 10.3390/cancers14081952

**Published:** 2022-04-13

**Authors:** Laura V. Bownes, Raoud Marayati, Colin H. Quinn, Andee M. Beierle, Sara C. Hutchins, Janet R. Julson, Michael H. Erwin, Jerry E. Stewart, Elizabeth Mroczek-Musulman, Michael Ohlmeyer, Jamie M. Aye, Karina J. Yoon, Elizabeth A. Beierle

**Affiliations:** 1 Division of Pediatric Surgery, Department of Surgery, University of Alabama at Birmingham, Birmingham, AL 35233, USA; lbownes@uabmc.edu (L.V.B.); rmarayati@uabmc.edu (R.M.); chquinn@uab.edu (C.H.Q.); abeierle@uab.edu (A.M.B.); jjulson@uabmc.edu (J.R.J.); mhe0004@uab.edu (M.H.E.); jessy@uab.edu (J.E.S.); 2 Division of Hematology and Oncology, Department of Pediatrics, University of Alabama at Birmingham, Birmingham, AL 35233, USA; schutchins@uabmc.edu (S.C.H.); jaimeaye@uabmc.edu (J.M.A.); 3 Department of Pathology, Children’s of Alabama, Birmingham, AL 35233, USA; elizabeth.mroczek-musulman@childrensal.org; 4 Atux Iskay LLC, Plainsboro, NJ 08536, USA; michael.ohlmeyer@gmail.com; 5 Department of Pharmacology and Toxicology, University of Alabama at Birmingham, Birmingham, AL 35233, USA; kyoon@uab.edu

**Keywords:** protein phosphatase 2A, neuroblastoma, patient-derived xenograft, CIP2A, MYCN

## Abstract

**Simple Summary:**

Despite significant advancements in neuroblastoma therapy, over half of the children with high-risk neuroblastoma will suffer disease relapse. Protein phosphatase 2A (PP2A) is a tumor suppressor that is decreased in neuroblastoma. Previous studies with FTY720, an immunomodulatory agent that activates PP2A, led to a decreased malignant phenotype in neuroblastoma. To activate PP2A without issues associated with the use of an immunomodulator, we investigated two novel PP2A-activating compounds, ATUX-792 and DBK-1154, designed for improved efficacy and fewer side effects. We aimed to demonstrate that ATUX-792 and DBK-1154 would decrease neuroblastoma cell survival and tumor growth with the goal of providing pre-clinical data to support advancing these compounds into the clinical arena.

**Abstract:**

Background: Protein phosphatase 2A (PP2A) functions as an inhibitor of cancer cell proliferation, and its tumor suppressor function is attenuated in many cancers. Previous studies utilized FTY720, an immunomodulating compound known to activate PP2A, and demonstrated a decrease in the malignant phenotype in neuroblastoma. We wished to investigate the effects of two novel PP2A activators, ATUX-792 (792) and DBK-1154 (1154). Methods: Long-term passage neuroblastoma cell lines and human neuroblastoma patient-derived xenograft (PDX) cells were used. Cells were treated with 792 or 1154, and viability, proliferation, and motility were examined. The effect on tumor growth was investigated using a murine flank tumor model. Results: Treatment with 792 or 1154 resulted in PP2A activation, decreased cell survival, proliferation, and motility in neuroblastoma cells. Immunoblotting revealed a decrease in MYCN protein expression with increasing concentrations of 792 and 1154. Treatment with 792 led to tumor necrosis and decreased tumor growth in vivo. Conclusions: PP2A activation with 792 or 1154 decreased survival, proliferation, and motility of neuroblastoma in vitro and tumor growth in vivo. Both compounds resulted in decreased expression of the oncogenic protein MYCN. These findings indicate a potential therapeutic role for these novel PP2A activators in neuroblastoma.

## 1. Introduction

Despite recent advancements in medical therapy, neuroblastoma, the most common pediatric extra-cranial solid tumor, is responsible for over 15% of pediatric cancer-related deaths [1]. Patients with high-risk disease have a high likelihood of disease recurrence with the existing standard of care therapy, which includes combination chemotherapy, surgical resection, stem cell transplant, radiation, immunotherapy, and retinoic acid differentiation [2]. These current therapies have adverse side effects that impose significant burdens even when life is extended [3]. Therefore, expanding our understanding of this disease and seeking new modes of intervention is crucial to develop more effective and less toxic therapies.

Protein phosphatase 2A (PP2A) is a serine/threonine phosphatase with known tumor suppressor function. The concept of therapeutic activation of PP2A in its role as a tumor suppressor has been in the literature for at least fifteen years [4], and there are two basic approaches to activation using small molecule therapeutics. The first involve targeting endogenously-expressed PP2A inhibitors, the most prominent cancerous inhibitor of protein phosphatase 2A (CIP2A) [5,6,7,8] and protein phosphatase-2A inhibitor-2, I2PP2A, (SET) [8,9,10]. Synthetic sphingolipids, of which the immunosuppressive compound FTY-720 is an example, binds SET, displaces it from PP2A complexes, and derepresses PP2A, thereby eliciting anti-tumor effects [11,12]. Our laboratory recently reported anti-tumor effects of FTY-720-induced PP2A activation in cellular and in vivo models of neuroblastoma [8] and medulloblastoma [13]. Direct allosteric binding and activation of PP2A by small molecules is a second therapeutic approach to potentiate the tumor suppressor function of PP2A. Investigation, and possible therapeutic exploitation, of this mechanism has recently become possible with the discovery that certain classes of tricyclic neuroleptics bind and activate PP2A [14,15]. One lead series resulting from medicinal chemistry on this class of compounds are the tricyclic sulfonamides [15]. These compounds act by binding PP2A AC heterodimers, resulting in activation of PP2A [16] via stabilization of classical PP2A ABC holoenzyme heterotrimers [17,18]. In the present study, we tested two direct PP2A activator compounds in neuroblastoma models, investigating their anti-tumor effects and mechanisms.

## 2. Materials and Methods

### 2.1. Cells and Cell Culture

We employed four neuroblastoma cell lines for these studies. The *MYCN* non-amplified cell line, SK-N-AS (CRL-2137, female), and the *MYCN* amplified cell line, SK-N-BE(2) (CRL-2271, male), were obtained from the American Type Culture Collection (ATCC, Manassas, VA, USA). SH-EP and WAC2, *MYCN* non-amplified and *MYCN* overexpressing isogenic cell lines, respectively (female), were a kind gift from M. Schwab (Deutsches Krebsforschungszentrum, Heidelberg, Germany) [19]. All cells were maintained with standard cell culture conditions at 5% carbon dioxide (CO_2_) and 37 °C. Dulbecco’s modified Eagle’s medium (DMEM, Corning Inc., Corning, NY, USA) with 10% fetal bovine serum (FBS, Hyclone, Suwanee, GA, USA), 1 μg/mL penicillin/streptomycin (Sigma Aldrich, Burlington, MA, USA), 4 mM L-glutamine (Thermo Fisher Scientific Inc., Waltham, MA, USA), and 1 μM non-essential amino acids was used to maintain SK-N-AS cells. A 1:1 mixture of minimum Eagle medium and Ham F-12 medium (Corning) with 10% FBS (Hyclone), 1 μg/mL penicillin/streptomycin (Sigma Aldrich), 2 mM/L l-glutamine (Thermo Fisher Scientific Inc.), and 1 μM/L non-essential amino acids was used for SK-N-BE(2) cells. Roswell Park Memorial Institute (RPMI) 1640 medium (10-040-CV, Corning) with 10% FBS (Hyclone), 1 μg/mL penicillin/streptomycin (Sigma Aldrich), and 2 mM/L l-glutamine (Thermo Fisher Scientific Inc.) was used to maintain SH-EP and WAC2 cells. All cell lines were verified within the last 12 months using short tandem repeat analysis (Genomics Core, University of Alabama at Birmingham (UAB), Birmingham, AL, USA) and were deemed free of Mycoplasma infection.

Two patient-derived xenografts (PDXs) were used in this study, COA6 and COA129, which have been previously described in detail [20,21]. These PDXs were generated at our institution under UAB Institutional Review Board (IRB) and Institutional Animal Care and Use Committee (IACUC) approved protocols (IRB-130627006, IACUC-009186, respectively). In brief, following written informed consent of each patient’s guardian and assent from each patient as appropriate, a fresh tumor specimen was obtained from surgical excision and temporarily placed in serum-free Roswell Park Memorial Institute 1640 (RPMI, 30–2001, ATCC) medium. For tumor implantation, mice were anesthetized with 3% inhalational isoflurane and 16 mm^3^ tumor pieces were placed into the subcutaneous space of the flank. Animals were housed in a pathogen free environment while being monitored routinely for overall health and tumor growth through weekly tumor volume measurements. Tumor volumes were calculated using the formula (width^2^ × length)/2. When tumor volumes met IACUC parameters, tumors were harvested. The tumors were dissociated using a Tumor Dissociation Kit (Miltenyi Biotec, San Diego, CA, USA) per manufacturer’s protocol. The dissociated tumor cells were cultured in neurobasal (NB) medium (Life Technologies, Carlsbad, CA, USA, USA) with the addition of B-27 without Vitamin A (Life Technologies), N2 (Life Technologies), l-glutamine (2 mM, Thermo Fisher Scientific Inc.), gentamicin (50 μg/mL, Thermo Fisher Scientific Inc.), amphotericin B (250 μg/mL, Thermo Fisher Scientific Inc.), epidermal growth factor (10 ng/mL, Miltenyi Biotec, San Diego, CA, USA, USA), and fibroblast growth factor (10 ng/mL, Miltenyi Biotec). Following dissociation, PDX cells were maintained at 37 °C with 5% CO_2_ overnight prior to utilizing for experiments. Routine real-time qPCR was performed to assess the percentage of human and mouse DNA contained in the PDXs to ensure that the tumors did not harbor murine contamination (TRENDD RNA/DNA Isolation and TaqMan QPCR/Genotyping Core Facility, UAB, Birmingham, AL, USA). All PDXs were verified within the last 12 months using short tandem repeat analysis (Genomics Core, UAB).

### 2.2. Reagents and Antibodies

The following primary antibodies were used for Western blotting: rabbit polyclonal anti-MYCN (9405S) and rabbit monoclonal anti-vinculin (E1E9V) from Cell Signaling (Danvers, MA, USA), rabbit polyclonal anti-CIP2A (ab99518) from Abcam (Cambridge, MA, USA), monoclonal mouse anti-β-actin (A1978) from Sigma Aldrich, and rabbit polyclonal anti-SET (55201-AP) from Proteintech (Rosemont, IL, USA). FTY720 was purchased from Sigma Aldrich.

### 2.3. Synthesis of DBK-1154 and ATUX-792

PP2A activators, DBK-1154 and ATUX-792 were synthesized as described in Ohlmeyer and Kastrinsky [16,22] (US patent number 9937180) for DBK-1154, and in Ohlmeyer and Zaware by a minor modification of the route in a published patent application US 2018-0251456 for ATUX-792. See Supplementary Data Figure S1 for compound characterization. The compounds were light sensitive; therefore, they were stored in the dark in dark containers, and experiments were conducted in a dark hood.

### 2.4. Microsome Stability Assays

Microsome stability assays were performed by Bioduro, LLC. Briefly, 2.5 µL (100 µM in DMSO) of test compounds are added to 197.5 µL of human, rat, or mouse microsome preparation (0.633 mg/mL in phosphate buffer) and mixed gently at 37 ^°^C. The reaction is started by adding 50 µL NADPH (5 mM in phosphate buffer) and mixing at 37 °C. The final reaction microsome concentration is 0.5 mg/mL. The reaction is sampled at 0, 5, 15, 30, and 60 min. Reaction aliquots are quenched by adding to 1:1 methanol–acetonitrile containing analytical standards and analyzed by liquid chromatography with tandem mass spectrometry (LC-MS/MS).

### 2.5. CYP-3A4 Inhibition Assays

Time-dependent CYP-3A4 inhibition assays were performed by Bioduro, LLC. In brief, the test compound is pre-incubated with human liver microsomes (0.25 mg/mL in reaction) and NADPH (0.5 mM in reaction) for 5, 30, and 60 min to allow metabolite formation. Midazolam, a known standard substrate of CYP-3A4, is added to give 2.5 µM reaction concentration, and consumption (microsome stability) of midazolam is assayed as above.

### 2.6. Immunoblotting

Following treatment, cells were lysed on ice using radioimmunoprecipitation (RIPA) buffer with phosphatase inhibitors (P5726, Sigma Aldrich), protease inhibitors (P8340, Sigma Aldrich), and phenylmethanesulfonylfluoride (PMSF, P7626, Sigma Aldrich) for 60 min. Cell lysates were centrifuged at 17,000 rpm for 30 min at 4 °C. Pierce™ BCA Protein Assay (Thermo Fisher Scientific, Inc.) determined protein concentration and proteins were separated by electrophoresis on sodium dodecyl sulfate polyacrylamide (SDS-PAGE) gels. Expected size of the targeted proteins was confirmed using molecular weight markers (Precision Plus Protein Kaleidoscope, Bio-Rad, Hercules, CA, USA). Antibodies were used according to the manufacturers’ suggested protocol. Immunoblots were developed with Luminata Classico or Crescendo Western horseradish peroxidase substrate (EMD Millipore, Burlington, MA, USA). Either anti-vinculin or anti-β-actin (as available from manufacturer) served as an internal loading control. We performed densitometry of Western blots using ImageJ software (Ver 1.49, http://imagej.nih.gov/ij (accessed on 9 February 2022).

### 2.7. Protein Phosphatase 2A (PP2A) Activation

SK-N-AS cells (1 × 10^6^) were treated with FTY720 (5 µM), 792 (10 µM), or 1154 (10 µM) for 24 h and lysed using HEPES (AC215001000, Thermo Fisher Scientific Inc.), MgCl_2_ (MX0045-4, EM Science, Gibbstown, NJ, USA), KCl (02-003-741, Thermo Fisher Scientific Inc.), PMSF (Sigma Aldrich), dithiothreitol (EC-601, National Diagnostics, Atlanta, GA, USA), and Igepal CA-630 (I7771, Sigma Aldrich) for 20 min on ice. Cell lysates were centrifuged at 17,000 rpm for 30 min at 4 °C. The commercially available PP2A Immunoprecipitation Phosphatase Assay Kit (17–313, EMD Millipore) has been used by other investigators to directly measure PP2A activity [23] and was utilized per manufacturer’s protocol to evaluate the activity of PP2A following treatment and compared to untreated cells. Experiments were completed at least in triplicate, and data reported as mean fold change ± standard error of the mean (SEM).

### 2.8. Cell Proliferation and Viability

CellTiter 96^®^ Aqueous One Solution Cell Proliferation assay (Promega, Madison, WI, USA) was used to examine proliferation, and alamarBlue^®^ assay (Thermo Fisher Scientific, Inc.) was used to evaluate viability. Cells, concentrations ranging from 5 × 10^3^ to 1.5 × 10^4^, depending on cell type due to wide variation in growth characteristics between established cell lines and PDX cells, were plated in 96-well plates and treated with increasing doses of 792 or 1154 for 24 h. CellTiter 96^®^ or alamarBlue^®^ dye (10 µL) was added for proliferation and viability assays, respectively. A microplate reader (Epoch Microplate Spectrophotometer, BioTek Instruments, Winooski, VT) measured absorbance at 490 nm for CellTiter 96^®^ and at 570 nm for alamarBlue^®^, using 600 nm as a reference. Lethal dose 50% (LD_50_) and half-maximal inhibitory concentration (IC_50_) values were calculated based on 24 h time points. Experiments were completed with at least three biologic replicates, and data reported as fold change ± SEM.

### 2.9. Flow Cytometry

To evaluate cell cycle, cells (1 × 10^6^) were plated and maintained in respective media with reduced FBS (4%) for cell synchronization. After 24 h, cells were returned to standard culture media and treated with 792 (SK-N-AS 10 μM; SK-N-BE(2), SH-EP, WAC2 15 μM) or 1154 (SK-N-AS, SK-N-BE(2) 10 μM; SH-EP, WAC2 15 μM) for 24 h (concentrations based upon calculated LD_50_ from viability assay). Cells were washed with phosphate-buffered saline (PBS) and fixed at 4 °C for 30 min with 100% ethanol. Following a second PBS wash, cells were stained with propidium iodide (PI, Invitrogen, Waltham, MA, USA), RNAse A (0.1 mg/mL, Qiagen, Germantown, MD, USA), and 0.1% TritonX (Active Motif, Carlsbad, CA, USA), and FACSCalibur^TM^ Flow Cytometer (BD Biosciences, Franklin Lakes, NJ, USA) was used for examination. FlowJo software (FlowJo, LLC, Ashland, OR, USA) was used for data analysis. Data reported as mean percentage cells in phase ± SEM.

Apoptosis was evaluated using the FITC Annexin V Apoptosis Detection Kit (BD Biosciences) according to manufacturer’s protocol. SH-EP cells (5 × 10^5^) were treated with either 792 or 1154 (10 µM) for 24 h. Cells were trypsinized, washed with PBS, centrifuged, and re-suspended in 100 μL of 1× binding buffer (0.1 M HEPES/NaOH, 1.4 M NaCl, 25 mM CaCl2, pH 7.4). Annexin V-FITC staining solutions were added, and cells were incubated for 15 min at room temperature (20–25 °C) in the dark. Cells were washed in 1 mL of 1× binding buffer, centrifuged, and re-suspended in 500 μL of 1× binding buffer. PI was added, and cells sat on ice for 1 h prior to flow cytometry. Unstained cells and cells stained with either PI or Annexin V alone were utilized for negative controls and compensation. The percent of Annexin V and/or PI positive cells was determined via flow cytometry using the Attune NxT Flow Cytometer (Invitrogen). Data are represented as percent cell population with Annexin V + PI− stained cells representing early apoptotic cells, Annexin V + PI + cells representing late apoptotic cells, cells not stained with Annexin V or PI representing live cells, and cells stained with PI only representing necrotic or dead cells. FlowJo software (FlowJo, LLC) was used for data analysis. Data reported as mean ± SEM.

### 2.10. Cell Motility

Since the established neuroblastoma cell lines grow as adherent cells, migration in SK-N-AS, SK-N-BE(2), SH-EP, and WAC2 cells was examined using monolayer wound healing (scratch) assay. Cells (5 × 10^4^) were plated in 12-well plates and treated for 24 h with 792 (SK-N-AS 10 μM; SK-N-BE(2), SH-EP, WAC2 15 μM) or 1154 (SK-N-AS, SK-N-BE(2) 10 μM; SH-EP, WAC2 15 μM); doses based upon previously determined LD_50_. Once cells reached 80% confluence, a sterile 200 μL pipette tip was used to make a standard scratch in the cell layer. Photographs of the plates were obtained at 0, 12, 24, and 36 h. ImageJ MRI Wound Healing Tool (http://imagej.nih.gov/ij/ (accessed on 14 October 2019) [24] quantified the open wound area, and data reported as fold change of the open area ± SEM. Invasion was evaluated with a modified Boyden chamber technique. The insert bottoms were coated with collagen type I (10 µg/mL, MP Biomedicals, Santa Ana, CA, USA). The insert tops were coated with a layer of Matrigel^TM^ (1 mg/mL, 50 μL, BD Biosciences). Cells were treated for 24 h with 792 (SK-N-AS 10 μM; SK-N-BE(2), SH-EP, WAC2 15 μM) or 1154 (SK-N-AS, SK-N-BE(2) 10 μM; SH-EP, WAC2 15 μM), plated (4 × 10^4^ cells), and allowed to invade through the membrane for 24 h. Insert membranes were fixed using 4% paraformaldehyde, stained with 1% crystal violet for 15 min, photographed, and invasion quantified with ImageJ (http://imagej.nih.gov/ij/ (accessed on 10 September 2019)). Invasion data were reported as mean percent membrane occupied by cells ± SEM. Experiments were completed with at least three biologic replicates.

### 2.11. Plasma Sample Collection from Mice

Blood aliquots were collected via cardiac puncture (300 µL) sampling from anesthetized mice in tubes coated with lithium heparin, mixed gently, then kept on ice and centrifuged at 2500× *g* for 15 min at 4 °C, within 1 h of collection. The plasma was harvested and kept frozen at −70 °C until further processing.

### 2.12. Quantitative Bioanalysis (Plasma)

The plasma samples were processed using protein precipitation and analyzed by LC-MS/MS. A plasma calibration curve was generated. Aliquots of drug-free plasma were spiked with the test articles at the specified concentration levels. The spiked plasma samples were processed together with the unknown plasma samples using the same procedure. The processed plasma samples were placed in the autosampler until the LC-MS/MS analysis, at which time peak areas were recorded, and the concentrations of the test articles in the unknown plasma samples were determined using the respective calibration curve. The reportable linear range of the assay was determined, along with the lower limit of quantitation (LLOQ).

### 2.13. Pharmacokinetics

Plots of plasma concentration of test articles, ATUX-792 and DBK-1154, vs. time are constructed and shown in Supplementary Figure S2.

### 2.14. Animal Statement

The UAB IACUC (IACUC-09064) approved all animal experiments, and the studies were conducted within institutional, national, and NIH guidelines and in compliance with the Animal Research: Reporting of In Vivo Experiments (ARRIVE) guidelines.

### 2.15. In Vivo Tumor Growth

SK-N-AS (1.8 × 10^6^) or SK-N-BE(2) (1.5 × 10^6^) cells in 25% Matrigel™ (BD Biosciences) were injected into the right flank of 6-week-old female athymic nude mice (Fredricks, Charles River, Wilmington, MA, USA) (*n* = 5 for SK-N-AS vehicle treated, *n* = 7 for SK-N-AS 792 or 1154 treatment and all SK-N-BE(2) experimental groups). Calipers were used to measure tumors three times per week, and volumes were calculated by the formula (width^2^ × length)/2, where width was the smaller measurement. After tumors reached a volume of 100 mm^3^, animals were randomized to three groups to receive 100 µL of either vehicle (N,N-dimethylacetamide (DMA, 271012, Sigma Aldrich) and Kolliphor^®^ HS 15 (Solutol, 42996, Sigma Aldrich)), 792 (50 mg/kg in DMA and Solutol), or 1154 (50 mg/kg in DMA and Solutol) twice daily by oral gavage; dosing based on previously published in vivo cancer models [22]. Animals were weighed weekly and were humanely euthanized in their home cages with CO_2_ and cervical dislocation when control tumors reached 2000 mm^3^; either 9 (SK-N-AS tumors) or 19 days (SK-N-BE(2) tumors) following treatment initiation, or when IACUC parameters were met. Power analysis was completed prior to the study.

### 2.16. Statistical Analysis

All in vitro experiments were performed with at least three biologic replicates. For PDX cells, biologic replicates were represented by PDX tumors from at least three different animals. Data reported as the mean ± SEM [25]. Statistical analysis was performed with GraphPad Prism 9 using ANOVA or Student’s *t*-test as appropriate, and statistical significance was defined as *p* ≤ 0.05. Inclusion and exclusion criteria and attrition were not applicable in our current study.

## 3. Results

### 3.1. Novel PP2A Activators

**Figure 1:**
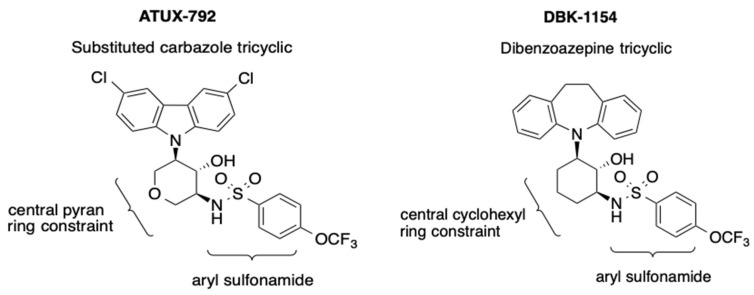
Tricyclic sulfonamides ATUX-792 (792) and DBK-1154 (1154). The compounds consist of a tricyclic moiety, a central ring constraint, and a pendant aryl sulfonamide. In 792 (***left panel***), the tricyclic is a substituted carbazole and in 1154 (***right panel***), the tricyclic is a dibenzoazepine.

Examples of the tricyclic sulfonamide PP2A activators 792 and 1154 are shown in Figure 1. The compounds consist of a tricyclic moiety, a central ring constraint, and a pendant aryl sulfonamide. DBK-1154 (1154) is the prototype in the series (*right panel*). The tricyclic in 1154 is a dibenzoazepine, and its major weakness is low stability with respect to oxidative metabolism, reflected in a short half-life and high clearance in liver microsome assays, as shown in Table 1A. The second compound, ATUX-792 (792) (Figure 1, *left panel*), is a more advanced member of the tricyclic sulfonamide PP2A activator type. Here, the tricyclic is a substituted carbazole and the central ring constraint is a pyran, rendering 792 stable with respect to oxidative metabolism, as shown in Table 1A. The unsubstituted carbazole is active in cell proliferation and PP2A activation; however, chlorine substitution enhances oxidative stability, which translates into low clearance in vitro (T_1/2_, Table 1A) and sustained plasma levels in vivo for 792 (Supplementary Figure S2). Compounds were assessed for time-dependent CYP-3A4 inhibition as shown in Table 1B. Treatment with 1154 showed a two-fold increase in CYP-3A4 inhibition after 60-min microsome pre-incubation and the inhibition was weak, 18 μM, indicating a low propensity to form CYP inhibitor metabolites. Treatment 792 showed no CYP-3A4 inhibition, and consequently no issue of time dependence or CYP inhibitor metabolite formation (Table 1B).

### 3.2. Treatment with 792 and 1154 Activated PP2A

Initially, nine PP2A activators in the tricyclic sulfonamide series were assessed in cell proliferation and viability using CellTiter 96^®^ or alamarBlue^®^ assays, respectively, in *MYCN* non-amplified SK-N-AS, and *MYCN* amplified, SK-N-BE(2), cells (Supplemental Figure S3). Compounds 792 and 1154 demonstrated the best effects on decreasing viability as well as proliferation (Supplemental Table S1). Based on these results, combined with in vitro microsome stability (Table 1 (A,B)) and in vivo exposure data (Supplemental Figure S2), we moved forward with investigating these two compounds.

**Figure 2:**
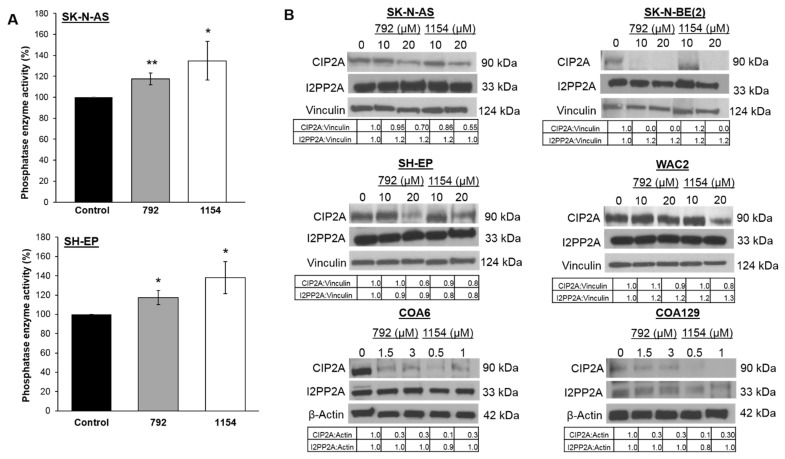
Effect of 792 or 1154 on PP2A activity. (**A**) Percent PP2A activity was measured following treatment with either 792 or 1154 (0, 10 μM for SK-N-AS, 15 μM for SH-EP) for 24 h in SK-N-AS (***upper panel***) and SH-EP (***lower panel***) cells. Both compounds increased PP2A activity significantly. (**B**) Immunoblotting was performed to detect protein levels of the endogenous PP2A inhibitors, CIP2A and SET. Whole cell lysates of four neuroblastoma long-term passage cells (SK-N-AS, SK-N-BE(2), SH-EP, WAC2) and two human neuroblastoma PDXs (COA6, COA129) treated with increasing concentrations of 792 or 1154 for 24 h were used. Expression of CIP2A was decreased following treatment with 792 or 1154 in all cell lines except in WAC2 and COA129 treated with 792. SET expression was unchanged. Data reported as mean fold change ± standard error of the mean (SEM), and experiments were repeated with at least three biologic replicates. Student’s *t*-test used for comparisons. * *p* ≤ 0.05, ** *p* ≤ 0.01, compared to control. The uncropped western blot figures were presented in Figure S7.

First investigations examined the ability of the compounds to activate the target, PP2A, using a PP2A immunoprecipitation phosphatase assay following treatment with 792 (10 μM) or 1154 (10 μM) for 24 h. PP2A activation significantly increased after treatment of SK-N-AS with 792 (117.5 ± 5.6% vs. 100 ± 0%, 792 vs. control, *p* ≤ 0.05, Figure 2A, *upper panel*) or 1154 (134.8 ± 18.4% vs. 100% ± 0%, 1154 vs. control, *p* ≤ 0.05, Figure 2A, *upper panel*). Similar results in PP2A activation were seen in the SH-EP cells treated with 792 (117.3 ± 7.2% vs. 100 ± 0%, 792 vs. control, *p* ≤ 0.05, Figure 2A, *lower panel*) or 1154 (138.0 ± 16.5% vs. 100 ± 0%, 1154 vs. control, *p* ≤ 0.05, Figure 2A, *lower panel*). Additionally, PP2A activation of FTY720 (5 μM) was examined for comparison. Compounds 792 and 1154 increased PP2A activation significantly more than FTY720 in SK-N-AS cells (*p* ≤ 0.01, Supplemental Figure S4). To determine if the expression of endogenous PP2A inhibitors, CIP2A and SET, were affected by 792 or 1154, immunoblotting was used. The expression of CIP2A was decreased following treatment with 792 or 1154 in all cell lines except in WAC2 and COA129 treated with 792. SET expression was unchanged (Figure 2B).

### 3.3. Treatment with 792 and 1154 Decreased Proliferation and Viability

**Figure 3:**
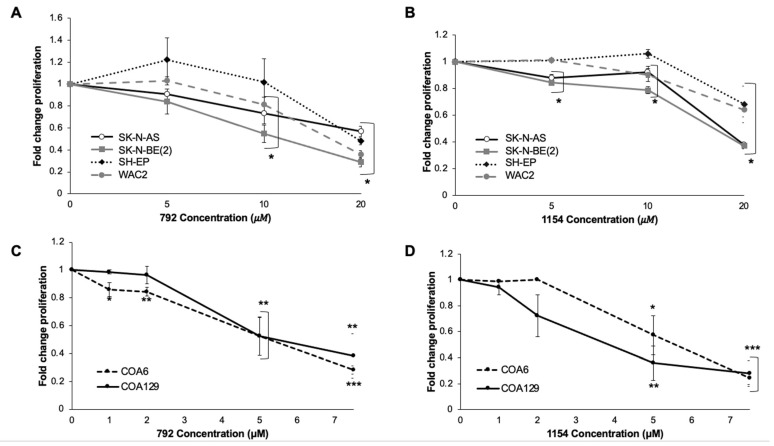
Treatment of neuroblastoma cells with 792 or 1154 resulted in decreased proliferation. Neuroblastoma cells (5 × 10^3^ for SK-N-AS, SK-N-BE(2), SH-EP, WAC2, COA6; 1 × 10^4^ for COA129) were plated in 96-well plates and treated with increasing doses of 792 or 1154 for 24 h. (**A**,**B**) Proliferation was significantly decreased in all long-term passage neuroblastoma cell lines following increasing concentrations of either 792 (**A**) or 1154 (**B**). (**C**,**D**) Increasing concentrations of either 792 (**C**) or 1154 (**D**) resulted in significantly decreased proliferation in the PDX cells. Data reported as mean fold change ± SEM, and experiments were repeated with at least three biologic replicates. Student’s t-test was used for comparisons. * *p* ≤ 0.05, ** *p* ≤ 0.01, *** *p* ≤ 0.001, compared to 0 μM.

We investigated the effects of 792 and 1154 on proliferation and viability in four long-term passage cell lines, SK-N-AS, SK-N-BE(2), SH-EP, and WAC2, as well as two patient-derived xenografts (PDXs), COA6 (*MYCN* amplified) and COA129 (*MYCN* non-amplified). Treatment with 792 and 1154 resulted in significantly decreased proliferation in the long-term cell lines (Figure 3A,B) as well as PDX cells (Figure 3C,D). Similarly, 792 and 1154 significantly decreased viability in all cell lines (Supplemental Figure S5A–D).

### 3.4. Treatment with 792 or 1154 Impaired Cell Cycle Progression

**Figure 4:**
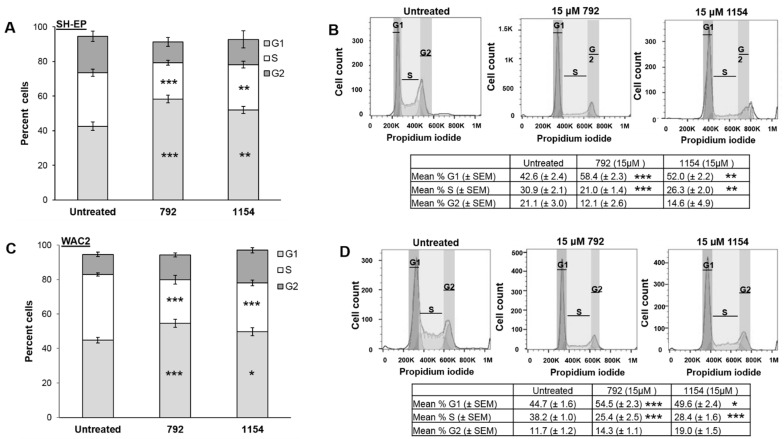
Compounds 792 or 1154 impaired progression through cell cycle. (**A**) Following treatment of either 792 or 1154 (0, 15 μM), SH-EP cells had increased percent cells in G1 phase and decreased percent in S phase compared to untreated cells, indicating a lack of cell cycle progression. Data reported as percent cells ± SEM. (**B**) Representative histograms of cell cycle analysis of SH-EP cells treated with either 792 or 1154 (0, 15 μM). Compiled data from at least three biologic replicates reporting the mean percent cells in phase ± SEM are presented in tabular form beneath the histograms. (**C**) WAC2 cells were treated with 792 or 1154 (0, 15 μM) for 24 h. WAC2 cells had significantly increased percentage of cells in G1 phase as well as decreased percentage in S phase indicating failure to progress through cell cycle. Data reported as percent cells ± SEM. (**D**) Representative histograms of cell cycle analysis of WAC2 cells treated with either 792 or 1154 (0, 15 μM). Compiled data from at least three biologic replicates reporting the mean percent phase ± SEM are presented in tabular form beneath the histograms. Experiments were repeated with at least three biologic replicates. Student’s *t*-test was used for comparisons. * *p* ≤ 0.05, ** *p* ≤ 0.01, *** *p* ≤ 0.001, compared to untreated (0 μM).

To further examine the decreased proliferation following treatment with 792 and 1154, the cell cycle was analyzed in the *MYCN* isogenic cell lines, SH-EP and WAC2, following treatment with 792 or 1154 (15 μM) for 24 h. Progression through the cell cycle was diminished in SH-EP cells, as demonstrated by a significant increase in the percentage of cells in G1 following treatment with either 792 (58.4 ± 2.3 μM vs. 42.6 ± 2.4 μM, treated vs. untreated, respectively, *p* ≤ 0.001, Figure 4A), or 1154 (52.0 ± 2.2 μM vs. 42.6 ± 2.4, treated vs. untreated, respectively, *p* ≤ 0.01, Figure 4A). The percentage of WAC2 in G1 was diminished following treatment with 792 (54.5 ± 2.3 μM vs. 44.7 ± 1.6, treated vs. untreated, respectively, *p* ≤ 0.001, Figure 4C) or 1154 (49.6 ± 2.4 μM vs. 44.7 ± 1.6 μM, treated vs. untreated, respectively, *p* ≤ 0.05, Figure 4). In addition, there was a decrease in the percentage of SH-EP cells in the S phase following treatment with either 792 (21.0 ± 1.4 μM vs. 30.9 ± 2.1 μM, treated vs. untreated, respectively, *p* ≤ 0.001, Figure 4A) or 1154 (26.3 ± 2.0 μM vs. 30.9 ± 2.1, treated vs. untreated, respectively, *p* ≤ 0.01, SH-EP, Figure 4A). The percentage of WAC2 cells in the S phase was diminished with 792 treatment (25.4 ± 2.5 μM vs. 38.2 ± 1.0, treated vs. untreated, respectively, *p* ≤ 0.001, Figure 4C) or 1154 (28.4 ± 1.6 μM vs. 38.2 ± 1.0 μM, treated vs. untreated, respectively, *p* ≤ 0.001, Figure 4C). Representative histograms for the cell cycles in SH-EP cells are presented in Figure 4B (*upper panel*), and for WAC2 cells in Figure 4D (*upper panel*). The compiled data from at least three biologic replicates are presented in tabular form beneath the histograms (Figure 4B,D, *lower panels*).

Initially, apoptosis was evaluated by examining the subG1 population in cell cycle analysis as previously described. Only SH-EP cells treated with 792 had a significant increase in the percent of cells in subG1 (Supplemental Figure S6A). Annexin V staining was performed to further examine apoptosis in SH-EP cells, and only treatment with 792 resulted in a significant increase in early apoptosis (Annexin V−  +  PI− cells) (Supplemental Figure S6B,C, *right lower quadrant*), whereas treatment with either 792 or 1154 led to increased cells in late apoptosis (Annexin V−  +  PI + cells) (Supplemental Figure S6B,C, *right upper quadrant*).

### 3.5. Cell Motility Decreased following Treatment with 792 and 1154

**Figure 5:**
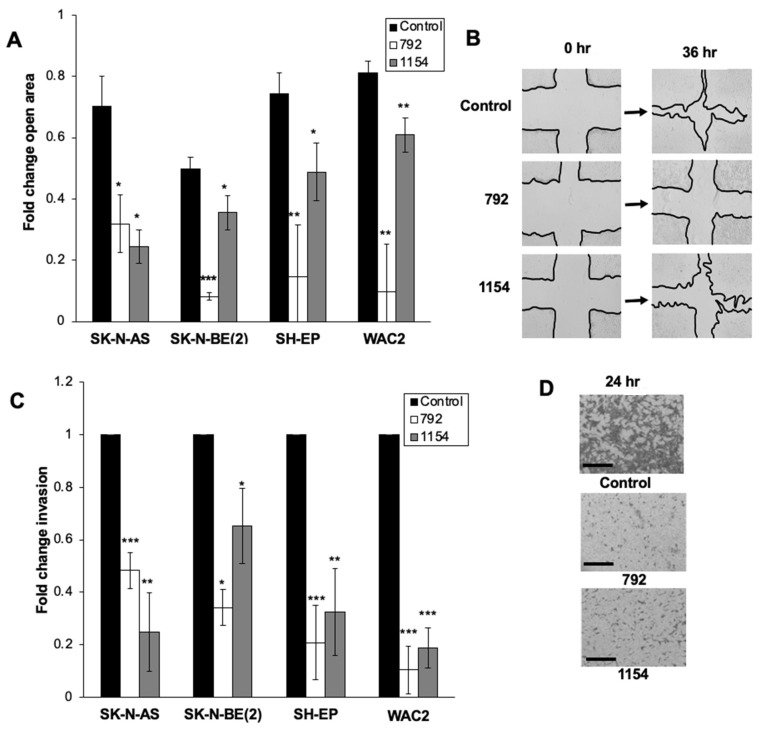
Treatment with 792 or 1154 decreased neuroblastoma migration and invasion. (**A**) For migration, neuroblastoma cells were plated and treated with 792 (SK-N-AS 10 μM; SK-N-BE(2), SH-EP, WAC2 15 μM) or 1154 (SK-N-AS, SK-N-BE(2) 10 μM; SH-EP, WAC2 15 μM). Cells were allowed to reach 80% confluence, and a standard scratch was made in the plates. Images of each well were taken at 0, 12, 24, and 36 h. The open wound area was analyzed using ImageJ. At 36 h of treatment, there was a significant decrease in cell migration compared to untreated cells. (**B**) Representative images of scratch assay at 0 and 36 h following treatment with 792 or 1154. (**C**) To evaluate invasion, neuroblastoma cells were treated for 24 h with 792 or 1154 at the same doses as scratch assay. After 24 h of treatment, cells were allowed to invade for 24 h through modified Boyden chamber membrane lined on top with a Matrigel^TM^ layer. Treatment with either 792 or 1154 resulted in significantly decreased invasion. Graph represents data reported as mean percent area of membrane with cells present ± SEM. (**D**) Representative images of stained invasion inserts demonstrating decreased number of invading cells following 24 h treatment with 792 or 1154. Data reported as mean ± SEM, and experiments were repeated with at least three biologic replicates. Scale bars represent 100 µm. Student’s *t*-test was use for comparison. * *p* ≤ 0.05, ** *p* ≤ 0.01, *** *p* ≤ 0.001, compared to control (0 μM).

Following treatment with 792 or 1154, cell migration was analyzed using a wound healing assay. Migration was significantly decreased in all four established neuroblastoma cell lines as shown by a decrease in the fold change of the open wound area after 36-h treatment with 792 (10 μM, SK-N-AS; 15 μM, SK-N-BE(2), SH-EP, and WAC2) or 1154 (10 μM, SK-N-AS, and SK-N-BE(2); 15 μM, SH-EP, and WAC2) (Figure 5A). Representative photographs of the wound at 0 and 36 h are shown (Figure 5B). Invasion was significantly decreased in the four cell lines following 24-h treatment with 792 or 1154 (Figure 5C,D). Representative pictures of the invasion inserts are shown (Figure 5D).

### 3.6. PP2A Activation Decreased Tumor Growth In Vivo

**Figure 6:**
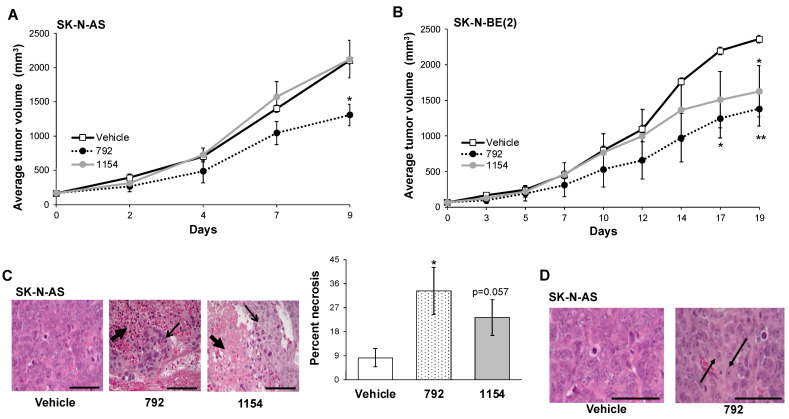
In vivo evaluation of 792 and 1154. SK-N-AS (1.8 × 10^6^) or SK-N-BE(2) (1.5 × 10^6^) cells were injected into the flanks of female athymic nude mice. Treatment with vehicle, 792, or 1154 (50 mg/kg, bid, po) began once tumors reached a volume of 100 mm^3^ (*n* = 5 for SK-N-AS vehicle treated, *n* = 7 for SK-N-AS 792 or 1154 treatment and all SK-N-BE(2) experimental groups). Tumor volumes were measured 3 times per week, and animals were weighed weekly. (**A**) Animals bearing SK-N-AS tumors treated with 792 had significantly decreased tumor volumes compared to those in animals treated with vehicle or 1154. Animals treated with 1154 did not show significant decrease in tumor volume compared to vehicle-treated animals. (**B**) In SK-N-BE(2) tumor-bearing animals, treatment with 792 and 1154 resulted in significantly decreased tumor volumes compared to vehicle. There was no significant difference in tumor growth in animals treated with 792 compared to those treated with 1154. (**C**) H&E staining revealed necrosis in 792- and 1154-treated tumors (*closed black arrows*). Areas of viable tumor are marked with open black arrows. Percent necrosis of the SK-N-AS tumors was quantified by a pathologist blinded to the treatment groups. Percent necrosis in SK-N-AS tumors from animals treated with 792 was significantly increased compared to tumors from animals treated with vehicle (33.3 ± 8.8% vs. 8.3 ± 3.3%, 792 vs. vehicle, respectively, *p* ≤ 0.05). Tumors from animals treated with 1154 also demonstrated increased necrosis but when quantified, did not reach statistical significance (23.3 ± 6.7% vs. 8.3 ± 3.3%, 1154 vs. vehicle, respectively, *p* = 0.057). (**D**) H&E staining of SK-N-AS tumors treated with 792 showed the presence of ganglion cells (*black arrows*), which were not seen in tumors from animals treated with vehicle. Presence of ganglion cells in neuroblastoma reflects tumor cell differentiation. Data reported as mean ± SEM. Scale bars represent 0.1 mm. Student’s t-test was use for comparison. * *p* ≤ 0.05, ** *p* ≤ 0.01, compared to vehicle.

Based on the results seen in vitro, we advanced investigations to an in vivo neuroblastoma flank model. SK-N-AS tumors were established in the right flank of athymic nude mice. Dosing of 792 and 1154 was based upon oxidative stability (Table 1A) and previous in vivo studies [22]. High clearance in mice (234 µL/min/mg, Table 1A) of 1154 prompted twice daily dosing at 100 mg/kg in previous studies to demonstrate anti-tumor effects [22]. For the current study, we chose to use the lowest possible dose and frequency of 1154 consistent with the probability of observing an in vivo effect. Therefore, we employed 50 mg/kg, *per os* (po), twice daily (bid), and ran 792 in direct comparison. Animals bearing SK-N-AS flank tumors treated with 792 (50 mg/kg bid, po) had significantly decreased tumor volumes after 9 days (1309 ± 169 mm^3^ vs. 2104 ± 53 mm^3^, 792 vs. vehicle, *p* ≤ 0.05, respectively, Figure 6A). Animals treated with 1154 did not show decreased tumor growth compared to vehicle (2124 ± 275 mm^3^ vs. 2104 ± 53 mm^3^, 1154 vs. vehicle, respectively, Figure 6A). Likewise, in established SK-N-BE(2) flank tumors, after 17 days of treatment, tumor volumes were significantly decreased with 792 treatment (1383 ± 243 mm^3^ vs. 2362 ± 53 mm^3^, 792 vs. vehicle, respectively, *p* ≤ 0.05, Figure 6B). SK-N-BE(2) tumor volumes were significantly decreased following treatment with 1154 (1628 ± 362 mm^3^ vs. 2362 ± 53 mm^3^, 1154 vs. vehicle, respectively, *p* ≤ 0.05, Figure 6B) by day 19. There was no significant difference in SK-N-BE(2) tumor growth between 792 and 1154 treated animals (Figure 6B). Hematoxylin and eosin staining of SK-N-AS tumors revealed necrosis in the tumors from animals treated with 792 and 1154 (Figure 6C, *closed black arrow*). Areas with normal tumor cells are marked with open black arrows. Tumor necrosis was quantified by a board-certified pediatric pathologist (EMM) blinded to the treatment groups. Tumors from animals treated with 792 had significantly more necrosis than those from vehicle treated animals (Figure 6C, *right panel*). Tumors from animals treated with 1154 tended to have increased necrosis but did not reach statistical significance compared to those from vehicle treated animals (Figure 6C, *p* = 0.057, *right panel*). Further inspection revealed that tumors from animals treated with 792 had ganglion cells present, which reflects tumor cell differentiation (Figure 6D, *black arrows*).

### 3.7. Treatment with 792 or 1154 Led to Decreased MYCN Expression

**Figure 7:**
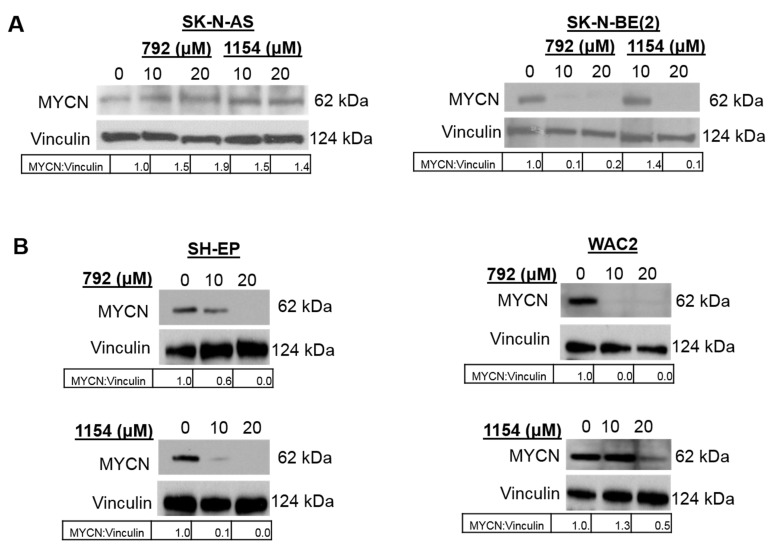
PP2A activation resulted in a decrease in MYCN expression. (**A**) Immunoblotting was completed on whole cell lysates of SK-N-AS, and SK-N-BE(2) cells following treatment with increasing doses of 792 or 1154 for 24 h. Treatment with 792 and 1154 resulted in decreased MYCN expression in SK-N-BE(2) cells (*right panel*), while there was an increase in MYCN expression in SK-N-AS cells (*left panel*). (**B**) Immunoblotting was completed on whole cell lysates of two isogenic *MYCN* neuroblastoma cell lines, SH-EP (*left panel*) and WAC2 (*right panel*). Treatment with 792 or 1154 resulted in decreased MYCN expression. Vinculin was used as a loading control, and densitometry was used to quantify MYCN expression relative to vinculin expression. The uncropped western blot figures were presented in Figure S7.

*MYCN* amplification is the most important negative prognostic indicator for neuroblastoma patient survival [1,2]. Other investigators have shown PP2A activation led to ubiquination and degradation of c-MYC, a member of the MYC transcription factor family with MYCN [26]. Therefore, we wished to investigate MYCN protein expression following treatment with 792 and 1154. After 24 h of treatment with increasing doses of 792 or 1154 (0, 10, 20 μM), SK-N-AS, SK-N-BE(2), SH-EP, and WAC2 cells were lysed for immunoblotting. Treatment with 792 or 1154 resulted in decreased expression of MYCN in SK-N-BE(2) (Figure 7A), SH-EP, and WAC2 (Figure 7B) cells.

## 4. Discussion

In this study, we utilized two novel tricyclic sulfonamides designed to activate PP2A and found that treatment with these compounds decreased neuroblastoma viability, proliferation, and motility in vitro and tumor growth in vivo.

PP2A functions as a tumor suppressor in numerous cancers, and its re-activation has been suggested as a potential therapeutic [8,13,14,16,27,28,29]. FTY720 (fingolimod) is a synthetic sphingolipid immunosuppressant that is FDA approved for the treatment of multiple sclerosis [30,31]. One of the primary effects of FTY720 is immunosuppression via functional S1PR inhibition [30], making evaluation of alternate PP2A activator chemotypes desirable.

The two tricyclic sulfonamides employed in the current study, 792 and 1154, are also referred to as small molecule activators of PP2A (SMAP) by other authors. These compounds act by binding PP2A AC heterodimers and facilitating the assembly of PP2A heterotrimeric holoenzymes [16,17]. In a recent report by Vervoort et al. [32], the Integrator-RNAPII complex was shown to associate directly with PP2A AC heterodimers, and this complex formation was potentiated by DBK-1154 [32,33]. This interaction with Integrator-RNAPIII provides a rationale for using tricyclic sulfonamides in cancer types where the transcriptional machinery has been dysregulated, i.e., transcriptionally addicted cancers [34], including neuroblastoma. For example, investigators showed that similar small molecule activators of this subunit resulted in decreased tumor growth in lung cancer [16,28,35]. Another study in glioblastoma found the prototype PP2A activator, DBK-1154, to decrease cell viability in vitro and increase survival in animals bearing intracranial glioblastoma tumors while demonstrating good tolerability and CNS exposure [22]. A second compound used in Merisaari et al. [22], NZ-8-061 (=DT-061), while active in vitro, showed significant time-dependent inhibition (>30 fold at 1 h with IC_50_ < 1 μM) of CYP3A4, which is indicative of the formation of CYP inhibitor metabolites and is a significant liability for drug–drug interactions and toxicity. This issue motivated our use of improved candidates, ATUX-792 and DBK-1154, in our present study that do not have this time-dependent CYP inhibition liability (Table 1B).

The synthetic sphingolipid FTY720 acts as a PP2A activator by binding the endogenous inhibitor SET and derepressing PP2A [36] Our lab has previously shown decreased neuroblastoma proliferation, motility, and in vivo tumor growth following small interfering RNA (siRNA) or short hairpin RNA (shRNA) knockdown of SET [8]. In the current investigation, we found that treatment with direct small molecule PP2A activators, 792 and 1154, had little effect on SET protein expression.

However, findings in this study on the effects of 792 and 1154 on the expression of another endogenous PP2A inhibitor, CIP2A, were different. Treatment with 792 or 1154 decreased CIP2A expression. CIP2A has been studied as a potential therapeutic target in several cancers, including neuroblastoma [8], colorectal cancers [5,37], renal cell carcinoma [38], and breast cancer [7]. Laine et al. recently demonstrated a decrease in CIP2A expression following treatment with the SMAPs, DBK-1154 and DT-061, in basal-like breast cancer [39], similar to the findings in the current study. Prior investigations from our lab found that treatment with the PP2A activator, FTY720, led to decreased CIP2A expression in two of the three medulloblastoma PDXs utilized [13], suggesting the effect of PP2A activation on CIP2A expression may be cell line specific. Other investigators propose that since MYCN and CIP2A are expressed during embryologic formation of the central nervous system, these two oncogenic proteins could be working together in neuroblastoma tumorigenesis [40]. Kerosuo et al. [40] demonstrated an interaction of these two proteins by investigating the knockdown of CIP2A and rescuing the neural crest phenotype by overexpressing MYCN. Of note is that CIP2A and MYCN expression were least affected in the *MYCN* non-amplified cell line, SK-N-AS, following PP2A activation, further suggesting a relation between CIP2A and MYCN, providing avenues for future investigations. Similarly, the increased expression of CIP2A and MYCN has been associated with a worse prognosis in neuroblastoma [40], making these compounds exciting therapeutics for a patient population in need of novel therapies.

PP2A has been well studied in its role in the cell cycle, and it is involved in numerous checkpoints with the majority of its dephosphorylation events negatively affecting cell cycle progression [41]. Cell cycle regulators are popular therapeutic targets in cancer [42]. Morita and colleagues investigated SMAPs in leukemia and found an increased percent of cells that were arrested in the G1 phase [18]. In the present study, we found a significant increase in the percent of cells in the G1 phase and a decrease in cells in the S phase following treatment with 792 or 1154 in the *MYCN* isogenic cell lines, SH-EP and WAC2, suggesting PP2A activation could be impairing cell cycle progression and that the effect may be MYCN independent. The ability of a cancer cell to avoid apoptosis or programmed cell death is an important mechanism driving tumor progression and growth; therefore, numerous cancer therapies are directed at activating the apoptotic pathway [43]. In lung cancer, SMAPs resulted in an increase in apoptotic markers and an increase in Annexin V staining [35]. Our lab has previously shown that treatment of hepatoblastoma cells with FTY720 resulted in an increase in cells in the subG1 phase, suggesting an increase in apoptosis following PP2A activation [44]. Other investigators also investigated FTY720 and found that treatment resulted in an increase in apoptosis of leukemia [45], glioma [46], and breast cancer [47] cells. We investigated the subG1 population of cells following 792 or 1154 treatment, and only SH-EP cells treated with 792 resulted in a significant increase in the percent of cells in subG1 (Supplemental Figure S6A). To further investigate apoptosis, we examined Annexin V and PI dual staining in SH-EP cells and found a significant increase in early apoptosis following 792 treatment and late apoptosis following treatment with either compound (Supplemental Figure S6B,C), confirming the increase in the subG1 population following 792 treatment. These findings suggest that apoptosis is likely not the primary mechanism driving the decrease in a malignant phenotype seen with these compounds and may be cell line and/or drug dependent.

*MYCN* is an oncogene that promotes cell proliferation, motility, and angiogenesis. Amplification of this oncogene is the most important negative prognostic indicator in neuroblastoma, and *MYCN* amplification automatically confers the patient into the high-risk treatment group [2,48]. Unfortunately, targeting MYCN as a therapeutic intervention has been difficult as the protein lacks enzyme activity and globular functional domains [49]. Investigators have been able to decrease MYCN expression using antisense oligonucleotides and showed decreased neuroblastoma proliferation in vitro and tumor growth in vivo [50]. Others have used DNA alkylation to target *MYCN* gene amplification to reduce the number of *MYCN* copies, which resulted in decreased neuroblastoma proliferation in *MYCN* amplified, but not *MYCN* non-amplified, cells [51]. Prior investigators have shown a relation between PP2A and c-MYC, demonstrating that PP2A dephosphorylates c-MYC at the serine 62 position, tagging c-MYC for ubiquitination and subsequent degradation [26]. In the current study, we show that PP2A activation resulted in decreased expression of the MYCN protein, independent of the *MYCN* amplification status of the tumor cells, suggesting a potential mechanism behind these compounds decreasing the malignant neuroblastoma phenotype and a potential therapeutic to target MYCN. It is known that the MYC family of transcription factors works in concert with RNAPII and the recently reported activation of INTS6/8-PP2A by 1154 [32], and the enhancement of promoter proximal pausing of RNAPII, may play a significant role in suppressing MYCN function, including the transcription of *MYCN*, by 1154 and 792. This mechanism will be the focus of future investigations.

Although treatment with 792 and 1154 decreased MYCN expression, we hypothesize that the lack of a larger effect on cell viability or tumor growth is likely due to the ability of cancer to proliferate via other oncogenic pathways, indicating a need for combination therapy. In hepatoblastoma, the PP2A activator, FTY720, was synergistic with the chemotherapeutic cisplatin, and combination therapy led to significantly decreased tumor volumes in vivo compared to either agent alone [44], suggesting a role for PP2A activation in combination with other agents. Similarly, synergy was found between FTY720 and the RTK/SRC/TEC inhibitor dasatinib in inhibiting pancreatic cancer proliferation [27]. DBK-1154 has been shown to enforce promoter proximal RNAPII pausing and the arrest of transcriptional elongation in cancer cells. The pharmacologically-activated Integrator-PP2A dephosphorylates the C Terminal Domain (CTD) of RNAPII, which counteracts phosphorylation by pTEFb, via its constituent kinase CDK9, which is known to drive RNAPII pause release and drive transcriptional elongation. Significant in vivo synergy in combination treatment has been observed between CDK9 inhibition and PP2A activation by DBK-1154 [32].

We investigated 792 and 1154 in an in vivo murine model and found that tumor growth was significantly decreased in both *MYCN* amplified and non-amplified tumors following treatment with 792, but 1154 significantly decreased tumor growth in only the *MYCN* amplified tumors. We hypothesize that these findings may be attributed to several factors. First, the increased metabolic stability and extended half-life of 792 gives higher systemic exposure after the oral dose compared to 1154 and may account for the improved effects on tumor growth. Second, SK-N-BE(2) neuroblastoma cells are *MYCN* amplified, and SK-N-AS cells are not. Comparing immunoblotting results, there was a more marked effect on MYCN expression in SK-N-BE(2) in comparison to SK-N-AS cells after 792 or 1154 treatment. It is possible that 792 and 1154 decreased tumor growth in the SK-N-BE(2) tumors because of the greater dependence of these cells on MYCN.

## 5. Conclusions

In the current study, we utilized two novel tricyclic sulfonamides, 792 and 1154, to activate the tumor suppressor, PP2A. Treatment was associated with the decreased survival, proliferation, and motility of neuroblastoma long-term passage and PDX cells in vitro. Treatment with both drugs resulted in decreased expression of the oncogenic proteins MYCN and CIP2A. In vivo, 792 had a greater effect on decreasing tumor burden compared to 1154 and resulted in ganglionic differentiation, suggesting a more mature tumor with a less aggressive phenotype post-treatment. These findings suggest a potential therapeutic role for these novel PP2A activators, particularly 792, in neuroblastoma and support the continued investigation to advance these compounds into the clinical arena to target a patient population in desperate need for novel therapies.

## Figures and Tables

**Table 1:** (**A**) Microsome (oxidative metabolism) stability studies revealed a short half-life (T_1/2_) and high clearance (CL) of 1154 in liver microsome assays. Longer T_1/2_ and lower CL renders 792 stable with respect to oxidative metabolism. (**B**) Studies of time-dependent inhibition of CYP-3A4 showed none associated with 792, and a moderate effect with 1154.

A. Microsome (Oxidative) Stability							
Tricyclic Class	Compound ID	T_1/2_ (min)			CL (µL/min/mg)		
		Mouse	Rat	Human	Mouse	Rat	Human
Subst. Carbazole	ATUX-792	>256	>256	>256	<5	<5	<5
Dibenzoazepine	DBK-1154	6	3	10	234	521	134
ID = Identification, T_1/2_ = Half-life, CL = Clearance, Subst.= Substituted							
**B. CYP-3A4 Time Dependent Inhibition**							
**Tricyclic Class**	**Compound ID**	IC_50_ * (µM) at Pre-incubation time				**IC_50_ Shift**	
**5 min**	**30 min**	**60 min**				**5/60 min**	
Subst. Carbazole	ATUX-792	>50	>50	>50		NA	
Dibenzoazepine	DBK-1154	42	27	18		2.4	

ID = Identification, Subst = Substituted, min = minutes. * Concentration required for 50% inhibition of midazolam (a CYP-A4 substrate) turnover.

## Data Availability

No date was reported in this study.

## Supplementary Materials

The following supporting information can be downloaded at: https://www.mdpi.com/article/10.3390/cancers14081952/s1, Figure S1: Atus-792 and DBK-1154 compound characterization; Figure S2: Bioavailability of ATUX-792 and DBK-1154; Figure S3: Novel PP2A activating compounds decreased neuroblastoma proliferation and viability; Figure S4: PP2A activation comparing 792, 1154 and FTY720; Figure S5: Treatment of neuroblastoma cell lines with 792 or 1154 resulted in decreased viability; Figure S6: 792 and 1154 treatment and apoptosis; Figure S7: Full Western blots. Table S1: Neuroblastoma cells had the highest sensitivity to 792 and 1154.
