# Supplementary material for: Pre-Clinical Study Evaluating Novel Protein Phosphatase 2A Activators as Therapeutics for Neuroblastoma
Source: Cancers (Basel). 2022 Apr 13;14(8):1952. doi: 10.3390/cancers14081952 (PMC9026148; doi:10.3390/cancers14081952)
Supplement: Supplementary file 1 [file cancers-14-01952-s001.zip › cancers-1649550 revised Supplemental figures and tables.pdf]

cancers MDPI

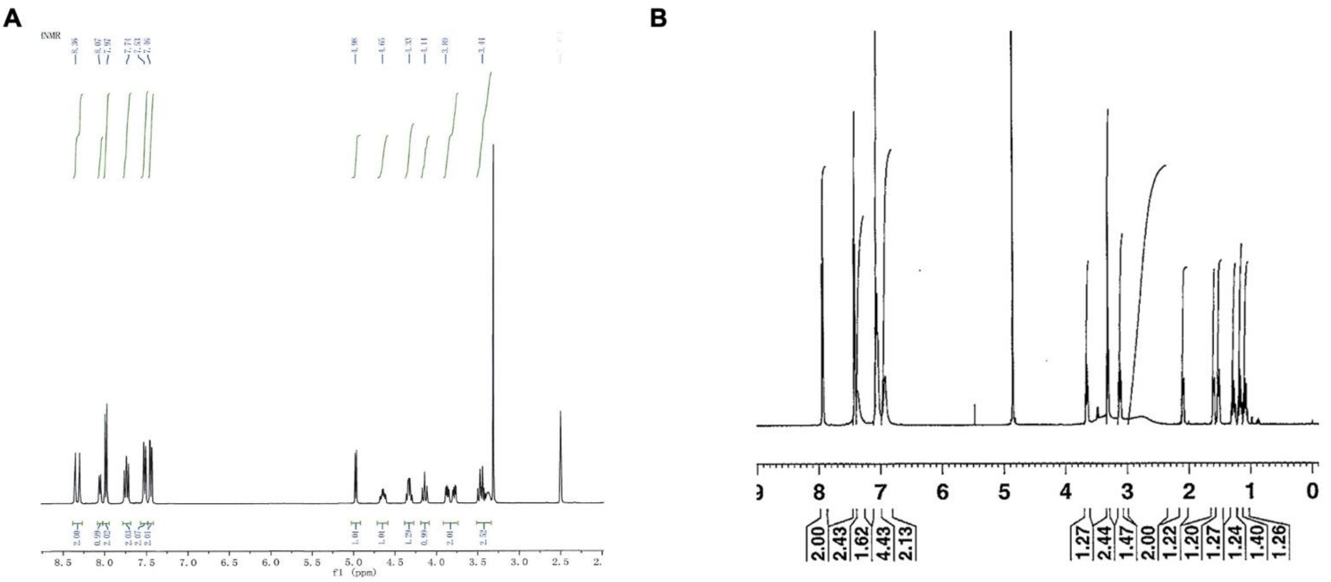

Supplemental Figure S1: Compound characterization for ATUX-792 and DBK-1154. (A) ATUX-792: N-((3S,4S,5R)-5-(3,6-dichloro-9H-carbazol-9-yl)-4-hydroxytetrahydro-2H-pyran-3-yl)-4-(trifluoromethoxy)benzenesulfonamide. 1H NMR (400 MHz, DMSO-d6) spectrum above; LCMS m/z 575.0369 ([M + H+], C24H20Cl2F3N2O5S requires 575.0417). Specific rotation [ ]D = -12.0º (c = 0.25, CH3OH). The enantiomeric identity, and purity was also confirmed by analytical chiral HPLC >99% (CHIRALPAK® OZ-H column, 70:30 hexanes-EtOH, 1.0 mL/min. (B) DBK-1154: N-(1S,2S,3R)-3-(10,11-dihydro-5H-dibenzo[b,f]azepin-5-yl)-2-hydroxycyclohexyl)-4-(trifluoromethoxy)benzenesulfonamide. 1H NMR (600 MHz, CD3OD) shown above; LCMS m/z 533.3625 ([M + H+], C27H27F3N2O4S requires 533.1716). Optically pure, >98%ee, DBK-1154 was obtained from Chiralcel OD-H resolution of racemete (Preparative Scale 80:20 hexane:iso-propanol) and Chiralcel IB-3 (Analytical Scale, 80:20 hexane:iso-propanol); specific optical rotation [ ]D = +16 (c = 1.0, EtOH).

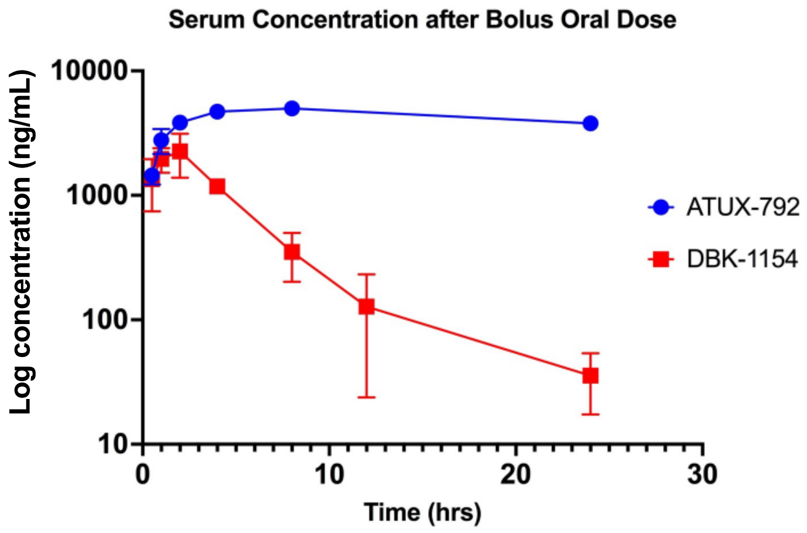

Animal Use	
Animal Species/Strain:	Mice / Male ICR
Animal Weight Range (gm):	20-30
Fasting Regimen:	4 hour fasting for PO group
Pre-dose Observations:	Body weight (gm)
**Test Article Dosing and PK Sample Collection**	
Test Article ID	ATUX-792 or DBK-1154
Route of Administration:	oral gavage
Dose Level (mg/kg)	50
Formulation (Vehicle), Concentration Test Article	10% Solutol/ 10% DMA/ 80% water, 6 mg/mL
Dose Volume (mL/mouse)	0.21 mL
Number of Animals per Time Point, n	n = 3
PK Sample (blood) Collection Times:	0.5, 1, 2, 4, 6, 8 and 24 hrs, ATUX-792<br>0.5, 1, 2, 4, 6, 8, 12 and 24 hrs, DBK-1154
Target Blood Sample Volume (mL):	No less than 0.3 mL via cardiac puncture sampling
Preferred Anticoagulant:	Lithium heparin
Preferred Sample Storage:	≤-70° C

Supplemental Figure S2: ATUX-792 has good oral bioavailability and sustained plasma levels compared to DBK-1154. Serum concentration after bolus oral dose of 50 mg/kg in 1:1:8 DMA:Solutol:water (3 animals per time point). Blood aliquots were collected via cardiac puncture (300 µL) sampling from anesthetized mice in tubes coated with lithium heparin, mixed gently, then kept on ice and centrifuged at 2,500 ×g for 15 min at 4°C, within 1 hour of collection. The plasma was harvested and kept frozen at -70 °C until quantitative bioanalysis. 1000 ng/mL = 2 mM for MW 500 g/mole. All aspects of this work including housing, experimentation, and animal disposal were performed in general accordance with the "Guide for the Care and Use of Laboratory Animals: Eighth Edition" (The National Academies Press, Washington, DC, 2011) in an AAALAC-accredited laboratory animal facility.

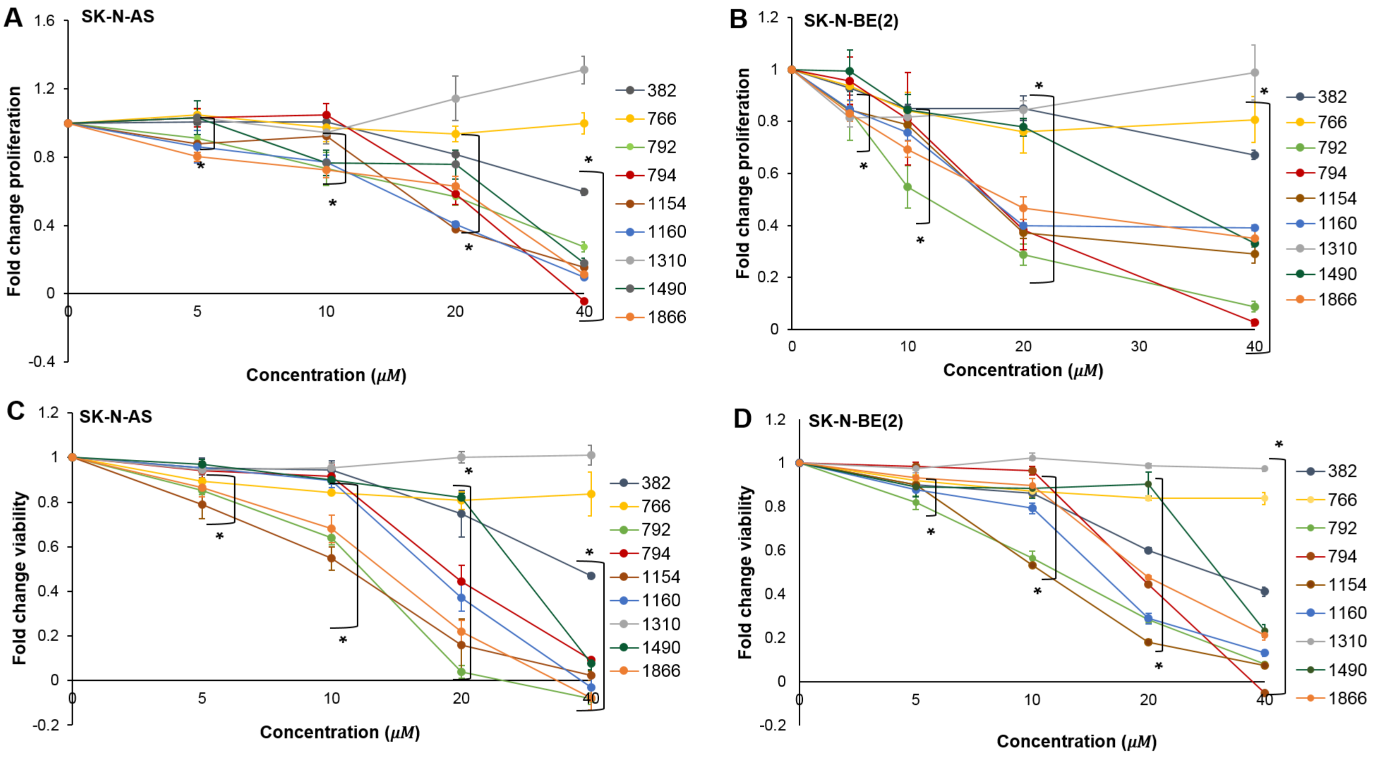

Supplemental Figure S3: Novel PP2A activating compounds decreased neuroblastoma proliferation and viability. SK-N-AS and SK-N-BE(2) cells (1.5 x 10^4) were plated in 96-well plates and treated with increasing doses of nine PP2A activating compounds for 24 hours. Proliferation was significantly decreased following treatment by a majority of the novel PP2A activators tested in (A) SK-N-AS and (B) SK-N-BE(2) cells. Similarly, PP2A activation using the novel activators resulted in decreased viability in (C) SK-N-AS and (D) SK-N-BE(2) cells. Data reported as mean fold change ± SEM, and experiments were repeated with at least three biologic replicates. Student's t-test used for comparisons. *p≤0.05, compared to 0 μM.

A

SK-N-AS

Drug	LD_{50} (μM)	SEM		Drug	IC_{50} (mM)	SEM
792	12.2	0.5		1160	20.4	1.0
1154	13.9	1.7		1154	22.4	0.4
1866	14.5	1.4		1866	22.5	1.4
1160	18.8	2.0		794	22.8	1.0
794	22.3	1.2		792	23.9	1.3
1490	25.0	0.9		1490	26.8	2.6
382	38.5	3.1		1310	59.3	17.0
766	81.0	9.4		382	60.8	2.4
1310	663.0	108.5		766	156.5	7.8

B

SK-N-BE(2)

Drug	LD_{50} (μM)	SEM		Drug	IC_{50} (mM)	SEM
1154	16.6	0.4		792	17.2	2.0
792	17.2	0.5		1160	17.2	0.2
1160	20.1	0.8		794	20.0	2.6
794	20.2	0.7		1154	23.8	1.4
1866	24.7	0.6		1866	25.9	1.4
1490	30.7	2.1		1490	31.9	1.6
382	32.0	1.3		1310	59.3	17.0
766	141.8	25.6		766	78.3	14.1
1310	853.3	93.3		382	132.9	6.8

Supplemental Table S1: Neuroblastoma cells had the highest sensitivity to 792 and 1154. **(A)** SK-N-AS and (B) SK-N-BE(2) cells (1.5 x 10^4) were plated in 96-well plates and treated with increasing doses of nine PP2A activating compounds for 24 hours. Compounds 792 and 1154 had the lowest LD_{50}s of the nine compounds investigated in both cell lines.

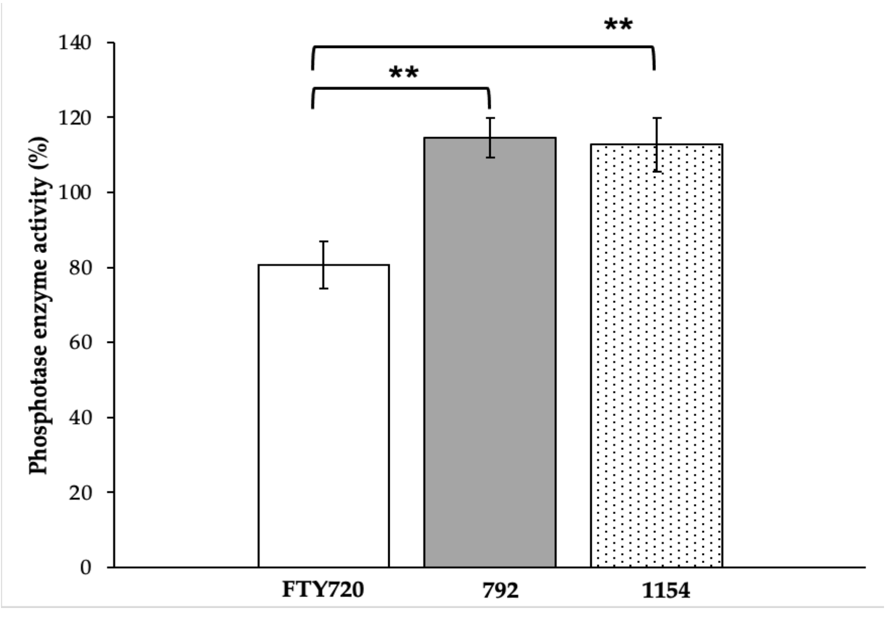

Supplemental Figure S4: SK-N-AS cells were treated with FTY720 (0, 5 μM), 792 (10 μM), or 1154 (10 μM) for 24 hours and PP2A activity was measured. Cells treated with 792 or 1154 had significantly increased PP2A activity compared to those treated with FTY720. **$p \leq 0.01$, compared to FTY720.

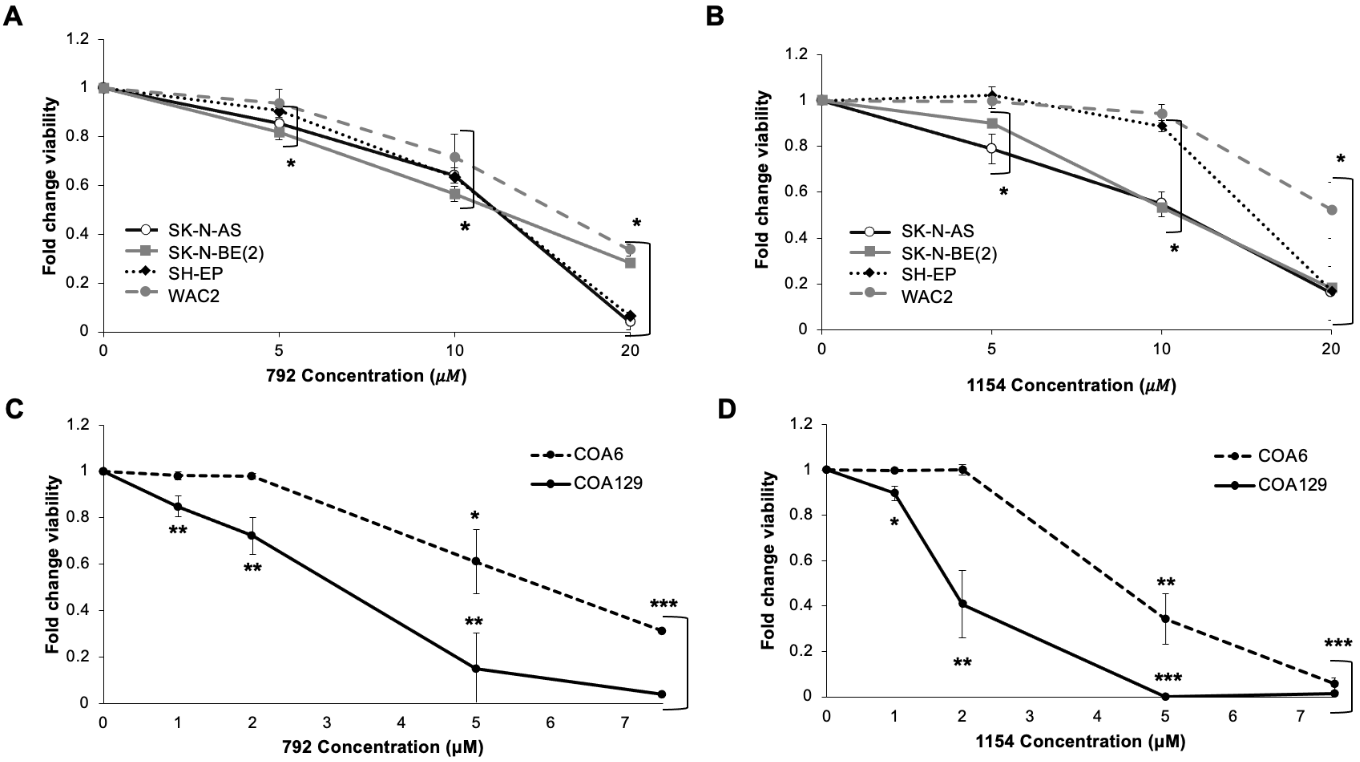

Supplemental Figure S5: Treatment of neuroblastoma cell lines with 792 or 1154 resulted in decreased viability. Neuroblastoma cells (1.5 x 10^4 for AS, BE, SH-EP, WAC, COA6; 3 x 10^5 for COA129) were plated in 96-well plates and treated with increasing doses of 792 or 1154 for 24 hours. All four long-term passage neuroblastoma cell lines showed decreased viability following increasing doses of either 792 (A) or 1154 (B). Similarly, increasing doses of either 792 (C) or 1154 (D) resulted in significantly decreased viability in the two PDXs. Data reported as mean fold change ± SEM, and experiments were repeated with at least three biologic replicates. Student's t-test used for comparisons. *$p \leq 0.05$, **$p \leq 0.01$, ***$p \leq 0.001$ compared to 0 μM.

A

Mean % <G1 (± SEM)	SK-N-AS	SK-N-BE(2)	SH-EP	WAC2
**Control**	3.9 ± 0.8	2.6 ± 1.2	2.3 ± 0.6	3.6 ± 0.7
**792**	4.2 ± 0.8	3.9 ± 0.9	6.3 ± 0.9 **	4.0 ± 1.1
**1154**	3.3 ± 0.5	4.3 ± 1.5	4.6 ± 0.2	1.7 ± 0.7

B

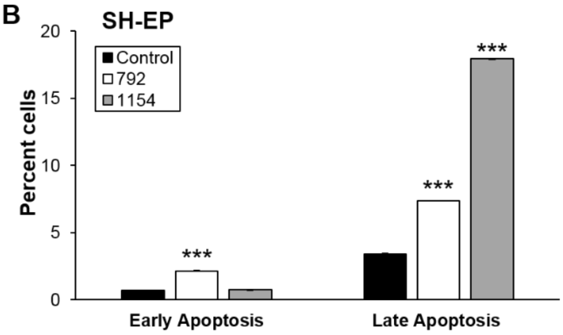

C

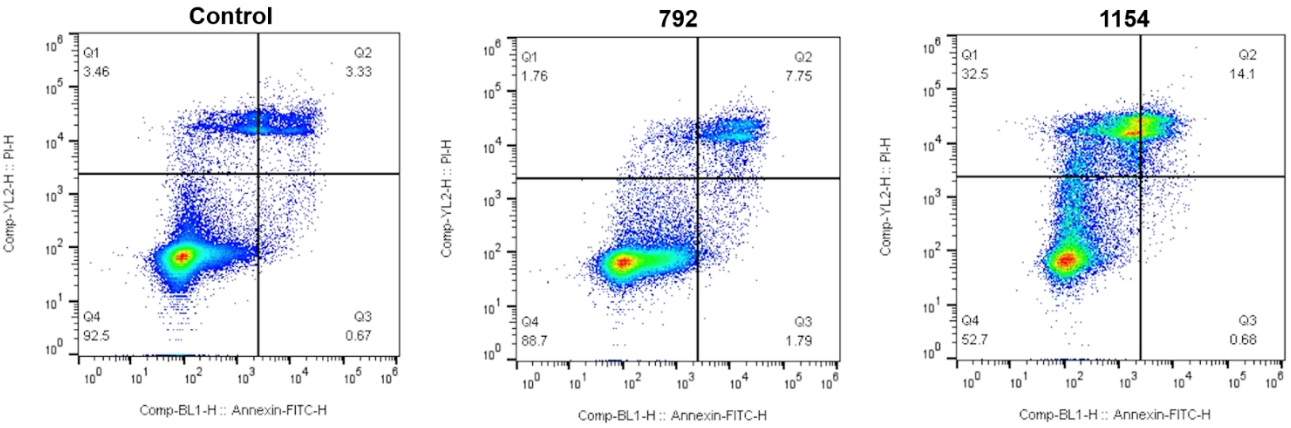

Supplemental Figure S6: 792 and 1154 treatment and apoptosis. Cells were serum starved and treated with 792 or 1154 to evaluate the cell cycle as previously described (Figure 4). (A) The percent of cells in the subG1 (<G1) is reported in tabular form. There was no significant increase in the subG1 population in any of the cell lines except for SH-EP cells following 792 treatment. (B, C) Because treatment of SH-EP cells resulted in an increased subG1 population, further investigation of apoptosis was performed. Cells were treated with 792 or 1154 (0, 10 µM) for 24 hours and were assessed by flow cytometry of Annexin V/PI dual staining, and FlowJo was used for analysis. Treatment with 792 resulted in a significant increase (B) in early apoptosis (Annexin V + PI- cells, quadrant Q3) (C) whereas treatment with 1154 only resulted in a significant increase (B) in late apoptosis (Annexin V + PI+ cells, quadrant Q2) (C). These data suggest that apoptosis is likely not the primary mechanism behind the effect of these drugs on viability. Data reported as mean fold change ± SEM. **p≤0.01, ***p≤0.001 compared to untreated.

Figure S7: Full Western blots

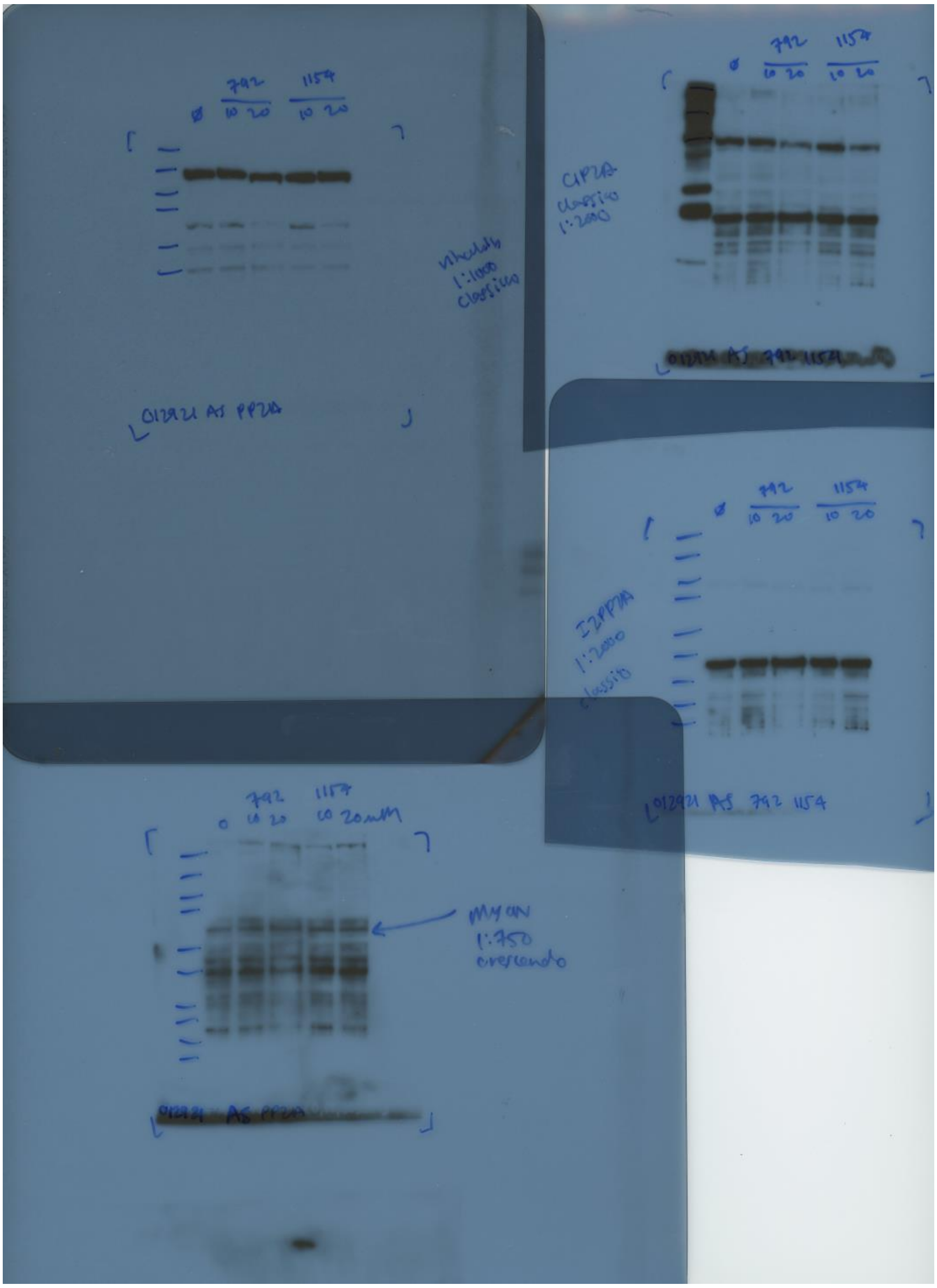

SK-N-AS: SET (I2PP2A), CIP2A, and Vinculin (Figure 2) and MYCN and Vinculin (Figure 7)

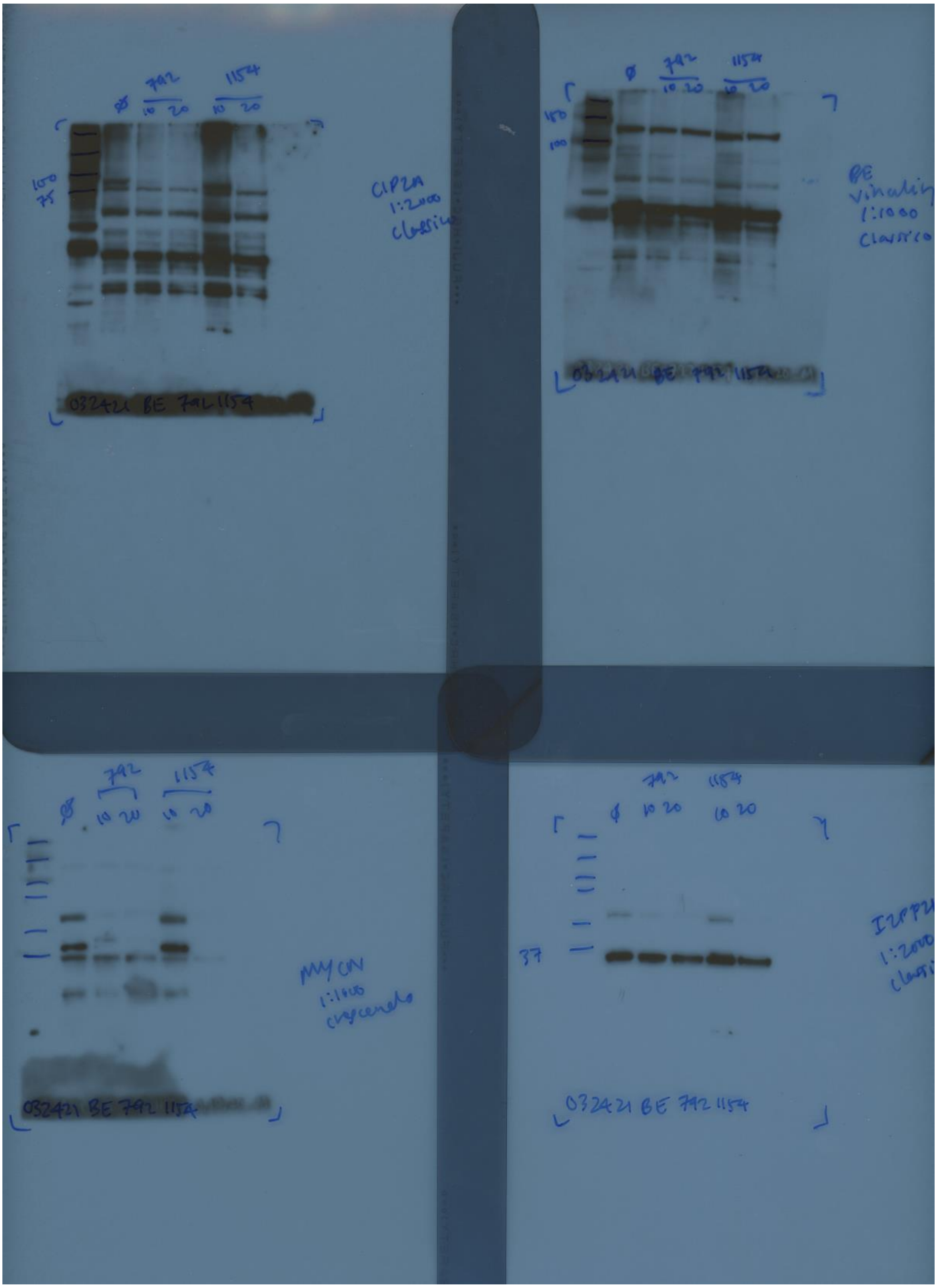

SK-N-BE(2): SET (I2PP2A), CIP2A, and Vinculin (Figure 2) and MYCN and Vinculin (Figure 7)

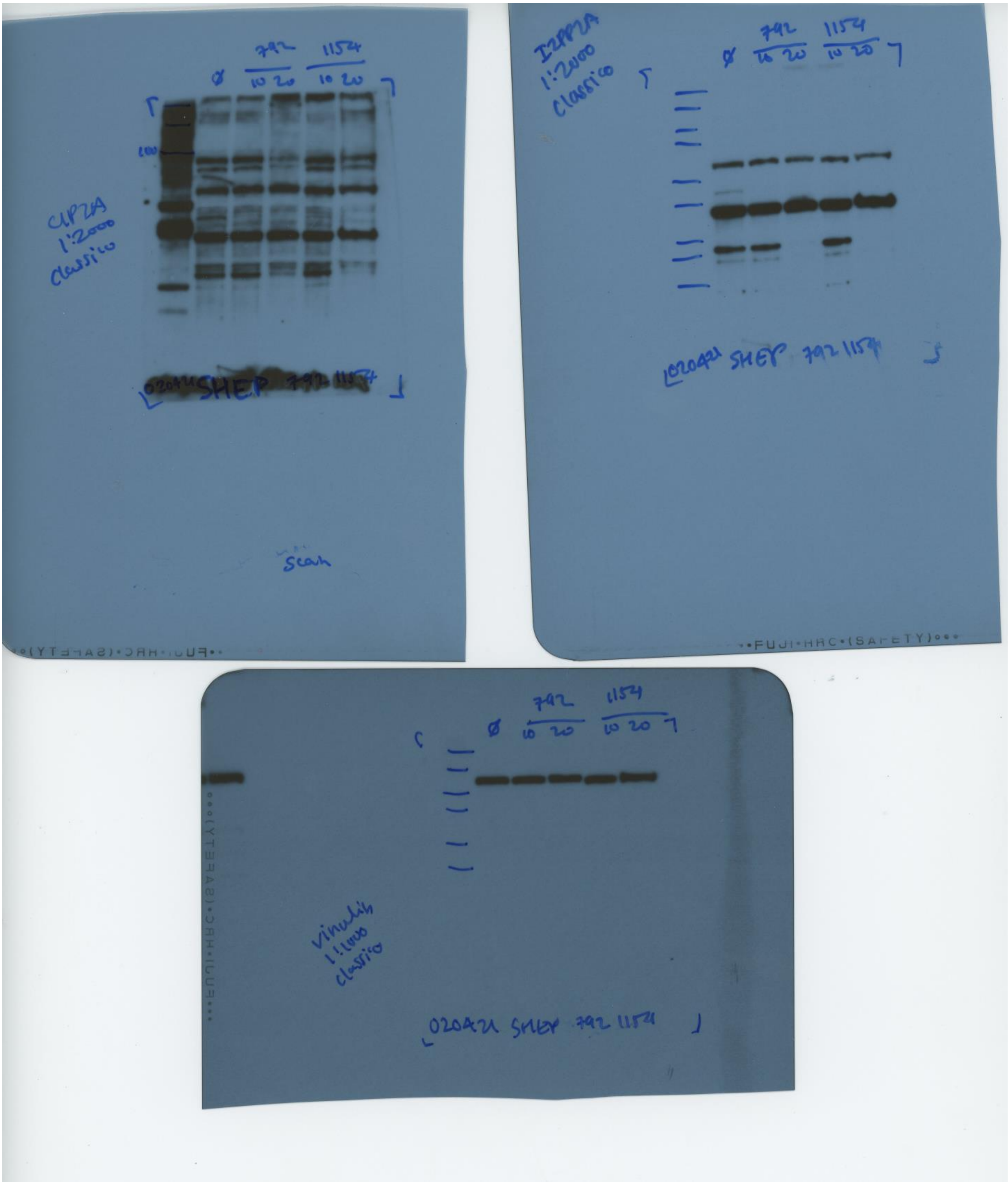

SH-EP: SET (I2PP2A), CIP2A, and Vinculin (Figure 2)

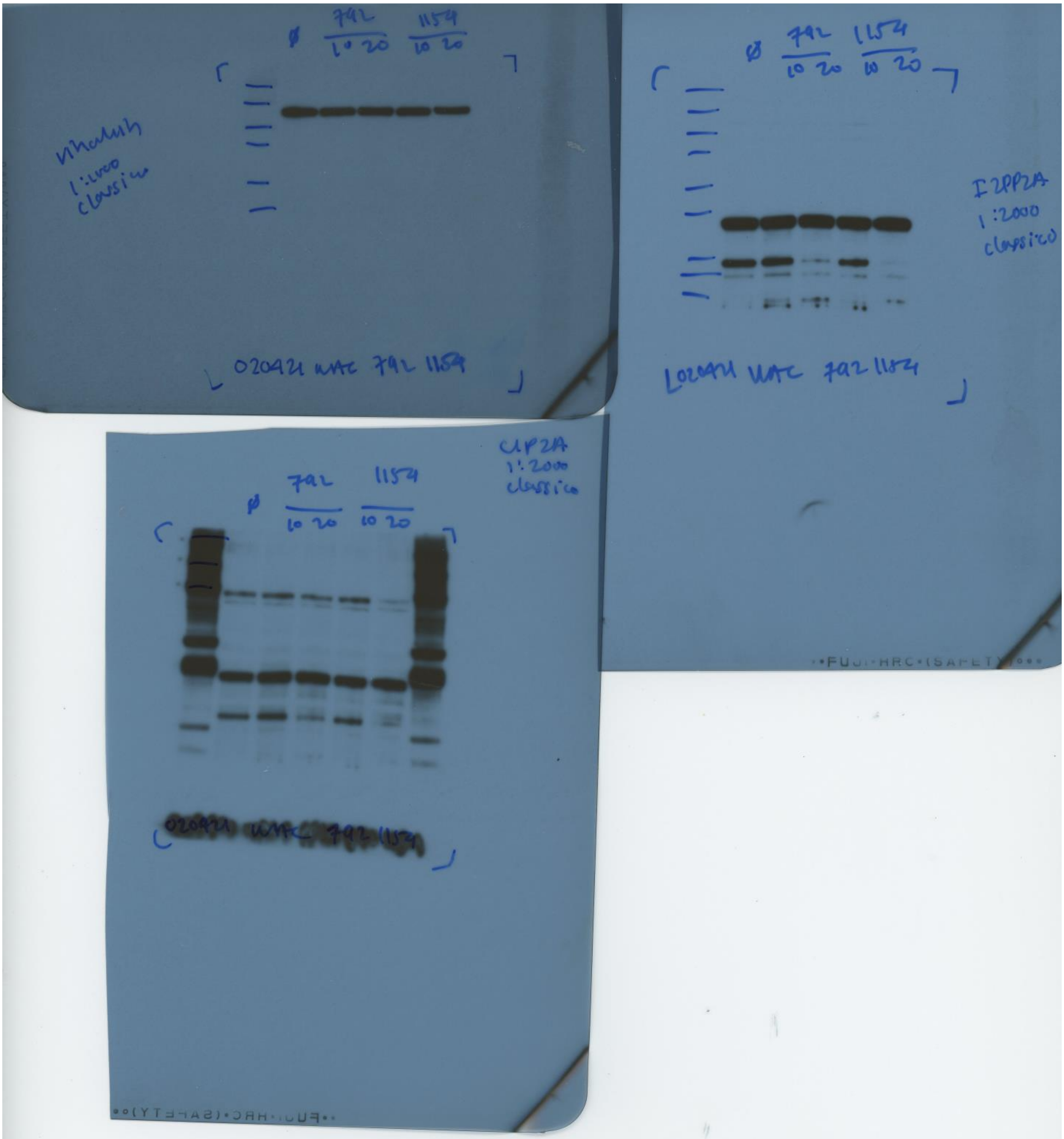
792
1154
10 20 10 20
020421 WAC 792 1154
I2PP2A
1:2000
classico
CIP2A
1:2000
classico
FUJI HRC (SAFETY)

WAC2: SET (I2PP2A), CIP2A, and Vinculin (Figure 2)

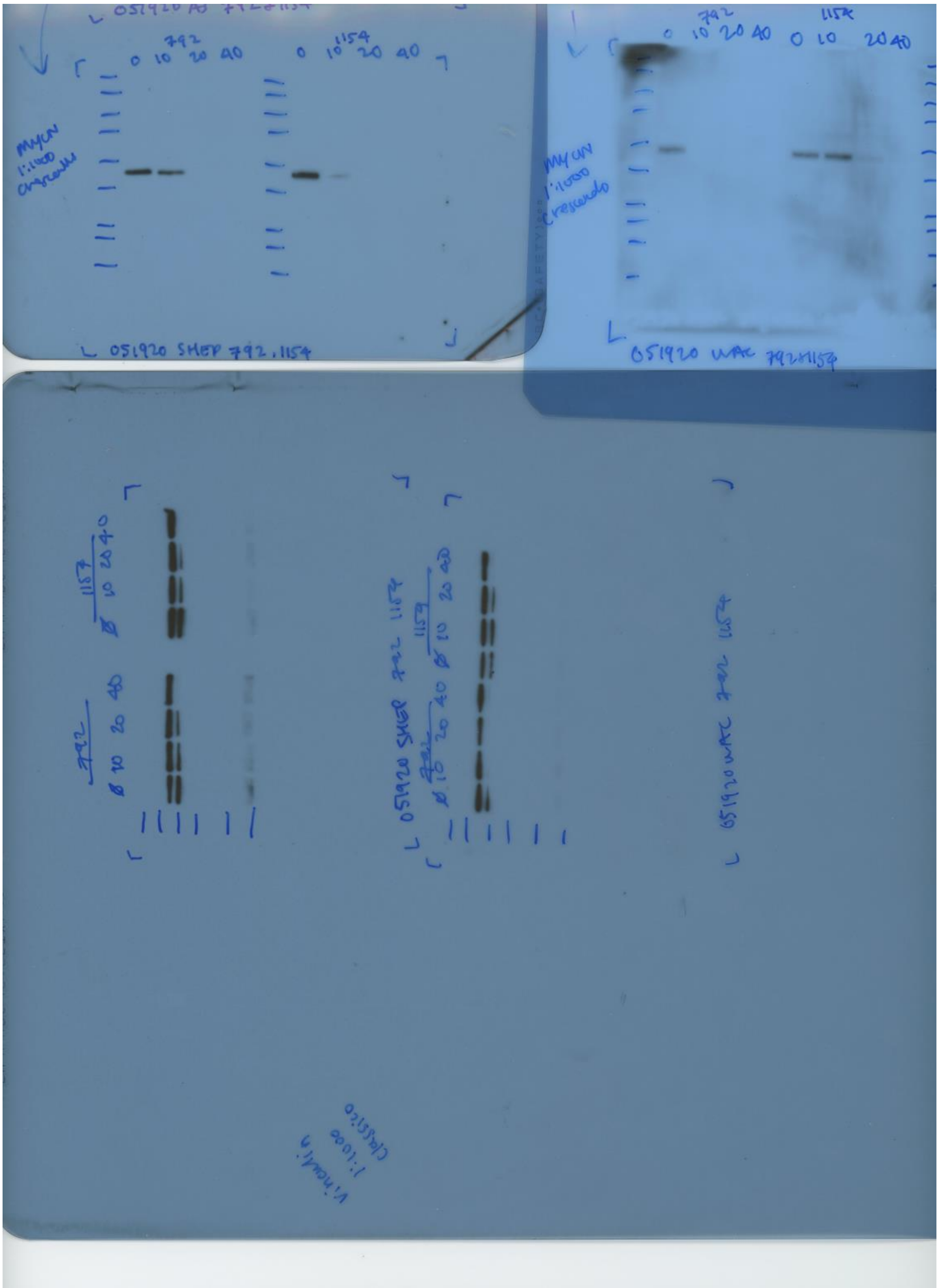

SH-EP and WAC2: MYCN and Vinculin (Figure 7)

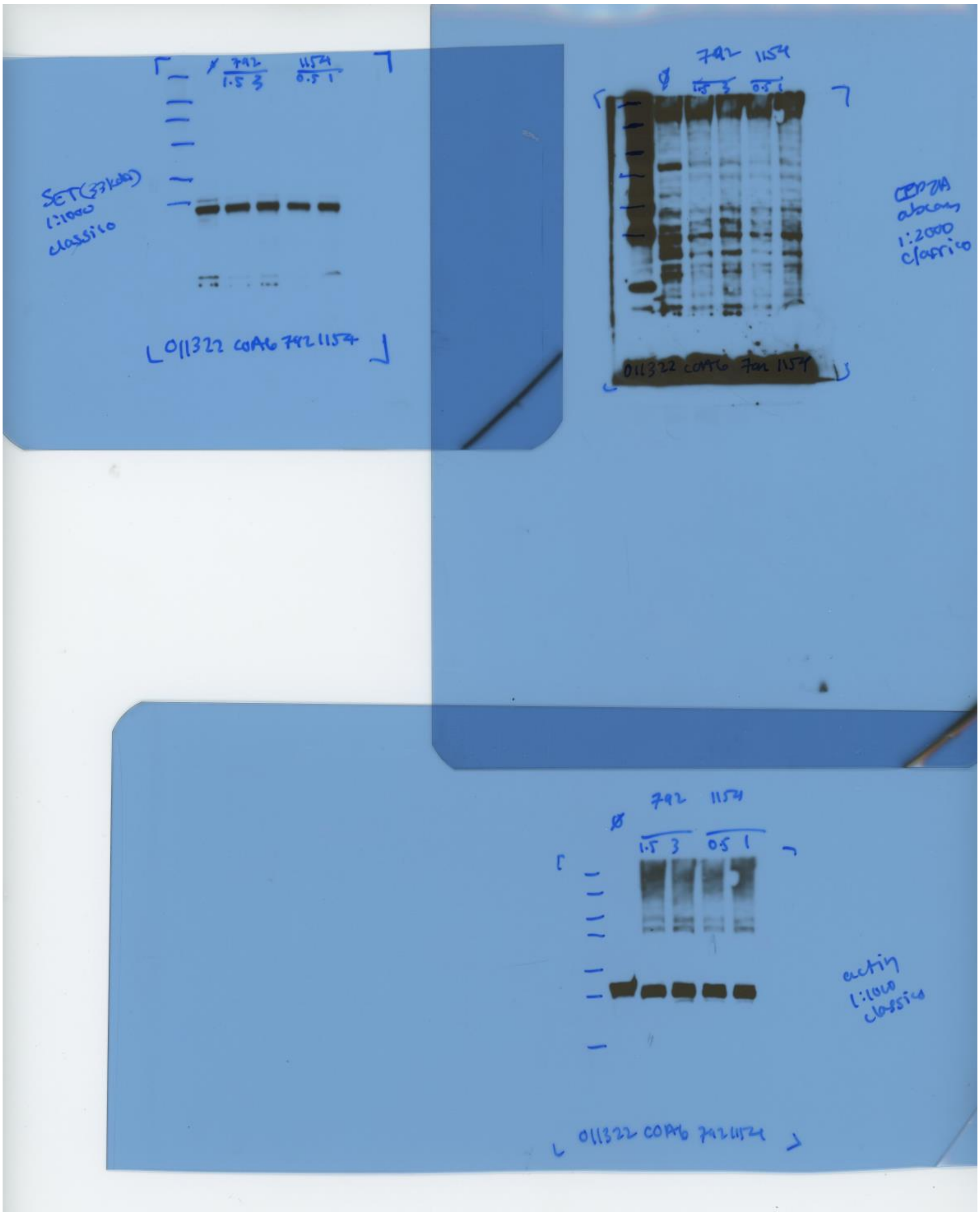

COA6: SET (I2PP2A), CIP2A, and Actin (Figure 2)

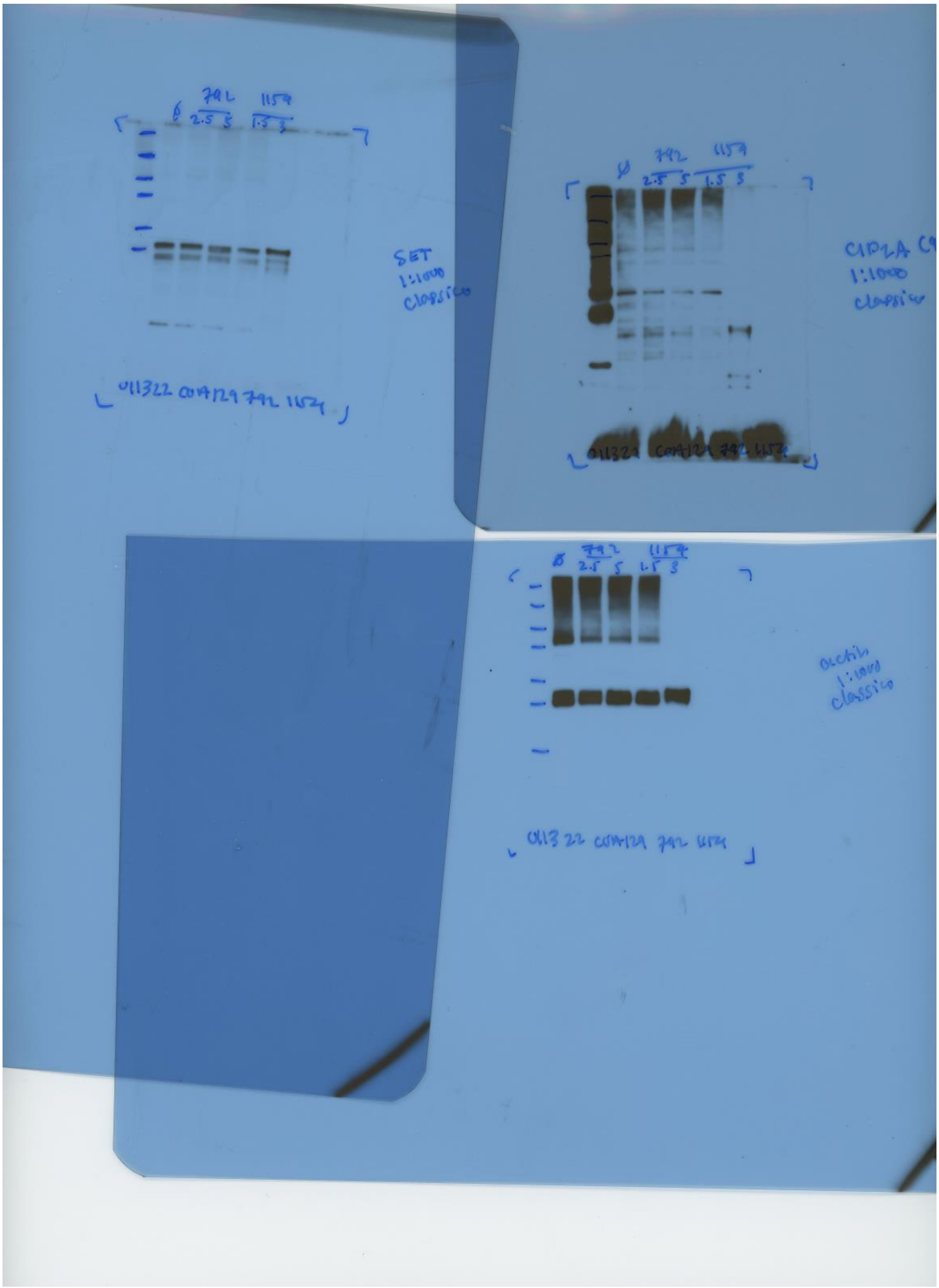

COA129: SET (I2PP2A), CIP2A, and Actin (Figure 2)
